# Outcomes and Trade-offs of Thailand’s 2022 Patient-Choice Dialysis Policy Reform

**DOI:** 10.1016/j.ekir.2026.106377

**Published:** 2026-02-25

**Authors:** Jeerath Phannajit, Kearkiat Praditpornsilpa, Kriang Tungsanga, Vuddhidej Ophascharoensuk, Talerngsak Kanjanabuch, Piyatida Chuengsaman, Chulathip Boonma, Kinanti Khansa Chavarina, Yot Teerawattananon, Wanrudee Isaranuwatchai

**Affiliations:** 1 Division of Clinical Epidemiology, Department of Medicine, King Chulalongkorn Memorial Hospital, Thai Red Cross, Bangkok, Thailand; 2 Division of Nephrology, Department of Medicine, Faculty of Medicine, Chulalongkorn University, Bangkok, Thailand; 3 Center of Excellence for Metabolic Bone Disease in CKD Patients, Faculty of Medicine, Chulalongkorn University, Bangkok, Thailand; 4 Bhumirajanakarindra Kidney Institute Hospital, Bangkok, Thailand; 5 Faculty of Medicine, Chiangmai University, Chiangmai, Thailand; 6 Center of Excellence in Kidney and Metabolic Disorders, Faculty of Medicine, Chulalongkorn University, Bangkok, Thailand; 7 Trinity Center-ISN Interventional Nephrology Training Center, Bangkok, Thailand; 8 Kiddee Kidney Care Peritoneal Dialysis Center, Bangkok, Thailand; 9 Health Intervention and Technology Assessment Program Foundation, Nonthaburi, Thailand; 10 Saw Swee Hock School of Public Health, National University of Singapore, Singapore, Singapore; 11 Institute of Health Policy, Management and Evaluation, University of Toronto, Toronto, Ontario, Canada

**Keywords:** dialysis policy, health system reform, hemodialysis, peritoneal dialysis, Thailand, universal health coverage

## Abstract

**Introduction:**

In February 2022, Thailand transitioned from a 14-year "peritoneal dialysis (PD)-first" policy to a "patient-choice" model, allowing unrestricted modality selection. This study evaluated the patient- and system-level impacts of this policy change on access, providers, expenditure, and outcomes.

**Methods:**

We linked nationwide administrative datasets (2018–2024). System-level trends in dialysis prevalence, incidence, and mortality were evaluated using interrupted time-series analysis. We further examined the program expenditure and shifts in care provision between the public and private sectors. For incident patients, we compared demographics, unplanned initiations, and hemodialysis (HD) vascular access use. All-cause mortality was modelled using multivariable Cox proportional hazards models, adjusting for key covariates.

**Results:**

Following the policy shift, dialysis utilization expanded rapidly; HD replaced PD as the dominant modality, driving increased incidence. Provision shifted to private providers, accompanied by more unplanned starts and prolonged use of temporary catheters. Expenditure increased by 74% from 2018, reaching USD 452 million in 2024. This accounted for 9.3% of the national health budget in a country with a GDP per capita of USD 7350. All-cause mortality increased, particularly within the first 90 days of treatment. Postpolicy initiation was associated with higher mortality, as were unplanned initiation, older age, and comorbidities.

**Conclusion:**

The reform resulted in rapid service expansion, requiring a substantially higher proportion of public funding and correlating with increased mortality. These findings underscore the importance of refining policy implementation strategies, including appropriate patient selection and implementing robust safeguards to ensure the integrity and sustainability of the national health system.

## Graphical abstract

**Figure:**
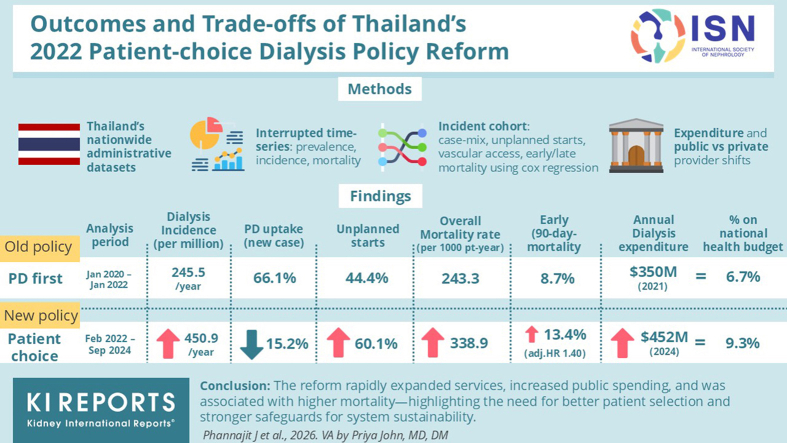

Kidney replacement therapy (KRT), including dialysis and kidney transplantation (KT) is a life-sustaining treatment with substantial resource requirements for treatment of people with kidney failure.^1^ KT offers the best quality of life, survival, and cost-effectiveness^2^^,^^3^; however, organ donation and access remain limited, especially in low- and middle-income countries, including Thailand.^4^ Consequently, most patients depend on dialysis, either HD or PD, to survive and maintain an acceptable quality of life. Without public financing, lifelong dialysis often results in catastrophic health expenditure,^5^^,^^6^ underscoring the need for universal health coverage to support KRT.

Thailand provided universal coverage for KRT in 2008 by introducing the “PD-first” policy under the Universal Coverage Scheme (UCS)—one of the 3 public health insurance schemes covering approximately 70% of the Thai population. This policy extended care to those previously excluded from preexisting schemes, which were restricted solely to government officials and corporate employees.^7^, ^8^, ^9^ To ensure equitable and sustainable access amid limited personnel and budgetary resources,^7^^,^^8^^,^^10^ the policy mandated PD as the default modality unless contraindicated.^11^ Following its implementation, the PD-first policy substantially expanded access to KRT, reduced out-of-pocket expenditures, and helped contain UCS budgetary growth. However, concerns regarding patient choice and inequity arose, because patients in other schemes retained the freedom to choose dialysis modality without restriction. Consequently, approximately 6000 (10% of patients under UCS dialysis) opted to pay out-of-pocket to access HD. These concerns generated growing political pressure on the Thai government, precipitating the calls for reform.^12^^,^^13^

On February 1, 2022, the Thai government, through the National Health Security Office (NHSO), which oversees the UCS, introduced a major reform allowing patients to choose their dialysis modality and extending coverage to previously self-financed HD.^14^ To facilitate rapid capacity expansion in response to the surging demand for HD, the reform involved a significant relaxation of quality assurance requirements for HD centers. Although aimed at promoting patient choice and reducing out-of-pocket costs, these changes carried risks of increased system burden, including a steep increase in HD utilization, pressure on service quality and expenditure, and a potential decline in PD to unsustainable levels. Furthermore, the rapid expansion of private-sector provision raised concerns about informal financial incentives and their potential to influence clinical decision-making and referral patterns.^15^

This study assessed the impact of Thailand’s 2022 dialysis policy using population-wide administrative data. We evaluated changes in dialysis prevalence, incidence, modality mix, provider landscape, public expenditure, and mortality; assessed shifts in patient characteristics and dialysis initiation pathways; and explored impacts on mortality outcomes. The findings provide important lessons for other low- and middle-income countries on designing balanced dialysis policies that strengthen capacity, equity, and sustainability; and offer evidence to guide future planning and resource allocation while maintaining an appropriated balance between sustainability, equity, and the provision of high-quality care.

## Methods

### Study Design, Data Sources, and Collaborations

This was a population-based observational study using linked national administrative datasets (2018–2024). Data sources (Supplementary Table S1), provided by the NHSO and the Thailand Renal Replacement Therapy Registry^16^ included claims databases, provider registries, budget reports, and vital status records. The claims databases captured real-time modality updates and session-level data entered by providers at the point of care. Detailed descriptions are provided in the Supplementary Methods and Supplementary Table S1. All analyses used deidentified data deterministically linked via encrypted identifiers. Data quality checks included deduplication, logical validation of timelines, and cross-verification of mortality records. Ethics approval was granted by the Institute for the Development of Human Research Protections (COA No. IHRP2024025).

This study was undertaken as part of a broader multistakeholder research initiative^17^ that brought together multimethod research and deliberation to develop relevant insights and recommendations to guide the future trajectory of this policy. The initiative operated through 3 pillars, namely an international learning committee of experts, a national working group appointed by the NHSO, and a research team led by the Health Intervention and Technology Assessment Program Foundation. The research team coordinated a multidisciplinary collaboration spanning health economics, statistics, policy, anthropology, and clinical nephrology. Throughout the process, 7 consultation rounds integrated perspectives from diverse stakeholders (including policymakers, clinicians, patients, and the private sector) to validate findings and develop final recommendations, which were submitted to the NHSO Board in November 2024.^17^

### Study Period and Populations

Analyses covered the period from January 1, 2018 to September 1, 2024, stratified into a prepolicy period (January 1, 2018 to January 31, 2022) and a postpolicy period (February 1, 2022 to September 1, 2024). Three complementary populations were defined to address specific objectives. First, a system-level aggregate dataset was constructed to assess longitudinal trends. This dataset comprised monthly counts of all prevalent patients alive on maintenance dialysis under UCS, as well as aggregate number of deaths, modality shifts, and KT events. Second, an incident dialysis cohort was identified comprising all patients initiating maintenance HD or PD between January 1, 2020 and September 1, 2024; this restricted timeframe was selected to ensure complete ascertainment of baseline characteristics and treatment data for the analysis of demographics, modality patterns, and survival. Third, a vascular access cohort included patients with HD established as their main modality at day 90 within this same 2020 to 2024 window to assess HD vascular access types and conversion; this definition excluded patients who switched to PD or received a transplant within 90 days. All eligible UCS records were included without a formal sample size calculation, and patients were followed-up with for vital status until December 31, 2024. Details of definitions are provided in the Supplementary Methods.

### Statistical Analyses

Data were extracted and transformed using Apache Hive SQL queried via DBeaver Community Edition v. 25.2.0 (DBeaver Corp, USA). Statistical analyses were conducted using Stata/SE 19.5 (StataCorp LLC, College Station, TX). Data visualization was performed using R version 4.5.1 (R Foundation for Statistical Computing, Vienna, Austria) with the ggplot2 package. All statistical tests were 2-sided with a significance threshold of *P* < 0.05; effect sizes were reported as 95% confidence intervals (CIs). No adjustments were made for multiple comparisons, and results were considered hypothesis-generating.

#### Interrupted Time-Series Analysis

The system-level policy effects were estimated using segmented interrupted time-series analysis spanning January 2018 to September 2024 (*n* = 81 months). Following established framework,^18^ we fitted generalized linear models with a negative binomial distribution and a logarithmic link function to account for overdispersed count data across all outcomes. The monthly metrics assessed included dialysis prevalence and incidence (per million population), KT incidence, modality shifts, and mortality.

The regression equation was specified as follows:

$$\ln\left(Y_{t}\right)=\beta_{0}+\beta_{1}\cdot {time}_{t}+\beta_{2}\cdot {policy}_{t}+\beta_{3}\cdot {time\_after}_{t}+\ln\left({exposure}_{t}\right)+\varepsilon$$

where $Y_{t\;}$ was the outcome count at month t . The policy variable was binary (1 from February 2022; 0 otherwise), ${time\_after}_{t}$ represented the number of months elapsed since policy implementation (coded 0 prior to February 2022), and ε represented the error term.

Exponentiated coefficients were presented as incidence rate ratios (IRR), with $\beta_{1}$ estimating the pre-policy baseline trend, $\beta_{1}$ the immediate change in level at the new policy implementation, and $\beta_{1}$ the change in post-policy slope. The midyear UCS beneficiaries was included as the exposure offsets (${exposure}_{t}$) for dialysis prevalence and incidence models, whereas the total prevalent dialysis population in each relevant modality was used as the exposure offset for models of modality shifts, transplantation, and mortality. Model fit and the choice of a negative binomial distribution were validated using the Likelihood Ratio test of the dispersion parameter, which confirmed overdispersion across all primary models (*P* < 0.001).

Model assumptions were rigorously verified using postestimation diagnostics. Serial correlation was assessed using the Cumby-Huizinga test on Anscombe residuals; no significant autocorrelation was detected for the first 10 lags (*P* > 0.05). As a robustness check, models were replicated using Newey-West standard errors to produce estimates robust to heteroscedasticity and potential autocorrelation. Seasonality was evaluated by including monthly indicator variables; though minor seasonal fluctuations were noted, they did not alter the significance of the primary policy indicators. Finally, national COVID-19 mortality peaks were identified to provide temporal context, but no statistical adjustments were applied for these surges.

#### Provider Distribution

Patient distribution across health care sectors was summarized monthly to assess shifts in the provider landscape. Providers were classified as public, private, or outsourced (private entities operating within public facilities) for HD, and as public or private for PD. Facilities offering both in-house and outsourced HD services were classified as outsourced because of data linkage limitations. To quantify the transition in service provision, we calculated the proportion of the total dialysis population treated by each provider type. Average proportions were compared descriptively between the prepolicy (January 2018–January 2022) and postpolicy (February 2022–September 2024) periods.

#### Financial Analysis

Annual fiscal data (2018–2024) were summarized to assess KRT budget allocation, actual expenditure, and share of total UCS spending (excluding provider salaries). Costs were specific to KRT (excluding nonerythropoietin medications and comorbidity management). Values are reported in THB and USD (1 THB = 0.2945 USD; September 1, 2024)^19^ without inflation adjustment. Fiscal years ran from October 1 of previous year to September 30 of the named year.

#### Changes in Patient Demographics and Dialysis Initiation Patterns

We compared baseline demographic and clinical characteristics between pre- and postpolicy incident cohorts, including age, sex, region, urbanicity (based on registration hospital location), and comorbidities identified via ICD-10 codes (Supplementary Table S2) and summarized using the Charlson Comorbidity Index (reported as mean ± SD and categories: 0–2, 3–4, 5–7, ≥8). Initial modality referred to the first treatment received, whereas the main modality at day 90 was defined for patients alive without transplant at 90 days (or the last modality for those who died within 90 days). Unplanned initiation was defined as starting KRT with HD via a nontunneled central venous catheter, including patients who subsequently transitioned to PD; conversely, all PD initiators who did not require temporary HD were classified as planned. Vascular access analyses focused on patients with HD as the main modality (the vascular access cohort), categorizing access as arteriovenous fistula/graft, tunneled, or non-tunneled catheter, with conversion time measured from initiation to the first permanent access use. To account for postpolicy adaptation, vascular access trends were presented annually. Categorical variables were reported as counts and percentages and compared using Pearson’s chi-square test. Continuous variables were reported as means ± SD or medians (interquartile range); differences between policy periods were assessed using *t* tests or Wilcoxon rank-sum tests, whereas yearly comparisons were evaluated using the Kruskal-Wallis test.

#### Policy’s Impact on Mortality Outcomes

System-level mortality trends were evaluated using the segmented interrupted time-series framework described previously. Mortality rates were calculated using the monthly system-level prevalent population as the denominator. Analyses were stratified by modality and timing: early mortality (occurring within 90 days of initiation) and late mortality (occurring at or beyond 90 days). To minimize bias from terminal transfers, deaths were attributed to a specific modality only if it had been maintained for ≥14 days before the event; otherwise, the death was assigned to the immediately preceding modality.

For the incident cohort, Cox proportional hazards models estimated mortality risk by policy period. Follow-up windows included the following: overall (day 0), early (0–90 days), and late (≥90 days, with a landmark at 90 days). Models were adjusted for unplanned initiation, age, sex, Charlson Comorbidity Index, and region. Proportional hazards assumptions were assessed using Schoenfeld residuals and log-minus-log plots. Follow-up ended at death, transplantation, the last claim, or December 31, 2024. Missing data across covariates were < 1%, and complete case analysis was applied.

## Results

### System-level Impact of the 2022 Dialysis Policy

**Figure 1:**
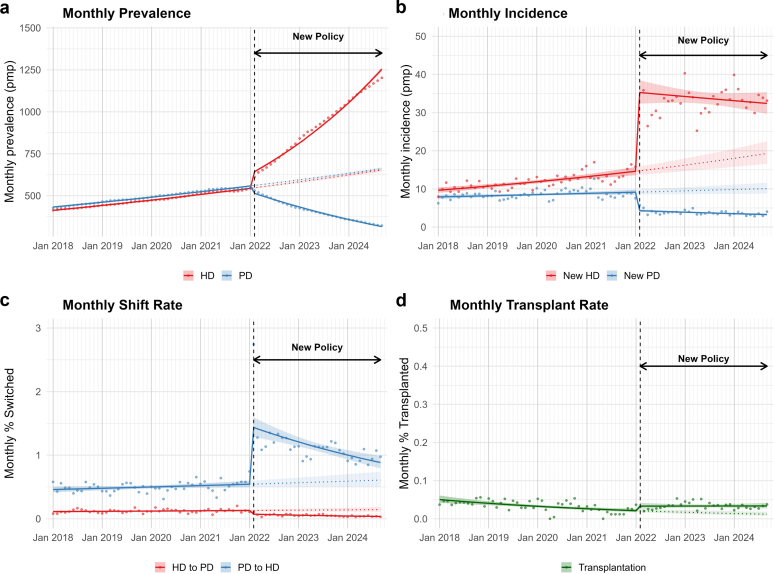
Trends in dialysis prevalence, incidence, modality shifts, and transplantation before and after the 2022 policy change. Monthly trends from January 2018 to December 2024 are shown for (a) prevalence of HD and PD (cases per million population [pmp]); (b) incidence of new HD and PD patients (pmp); (c) modality shift rates (% of active patients switching between HD and PD); and (d) kidney transplantation rates (% of active dialysis patients transplanted). Solid lines indicate fitted trends from segmented negative binomial regression models with 95% confidence intervals (shaded). Dotted lines show counterfactual trends without the policy. The vertical dashed line marks policy implementation in February 2022. HD, hemodialysis; PD, peritoneal dialysis.

The new policy precipitated a significant immediate surge in overall dialysis incidence (IRR: 1.67, 95% CI: 1.50–1.87), with the mean annualized rate rising substantially from 245.5 to 450.9 per million population (Supplementary Table S3). This aggregate increase was driven by a >2-fold increase in HD initiation and a simultaneous precipitous decline in PD initiation (Figure 1a and Supplementary Table S3). Consequently, the total active dialysis population expanded from approximately 52,000 to 72,000 patients by September 2024 (Supplementary Figure S1). Within this growth, HD prevalence exhibited a significant immediate level increase followed by sustained monthly expansion, significantly surpassing PD, which declined to <15,000 patients amidst a continuing negative trend (Figure 1b, Supplementary Table S3 and Supplementary Figure S1). This structural shift was further compounded by an increase in established patients switching from PD to HD at more than double the prepolicy rate (Figure 1c). Finally, though the analysis detected a significant increase in the transplantation rate and system volume, the rapid expansion of the dialysis population resulted in the individual probability of receiving a transplant remaining stagnant at a low rate of 0.4 events per 100 patient-years (Figure 1d and Supplementary Table S3).

In addition, the policy precipitated a restructuring of service provision. Publicly provided PD, formerly the backbone of the system, declined from 49.0% to 27.8% of the total dialysis population. Conversely, HD provision expanded across all sectors. This growth was most pronounced in private centers, where the share of the total patient population increased from 27.7% to 40.0%. Outsourced units (private providers operating within public facilities) also increased their share from 14.0% to 19.2%, whereas public hospitals saw a modest increase from 8.8% to 12.9%. Collectively, these shifts mark a definitive transition from a system dominated by public PD provision to one driven by private HD services (Supplementary Figure S2).

**Figure 2:**
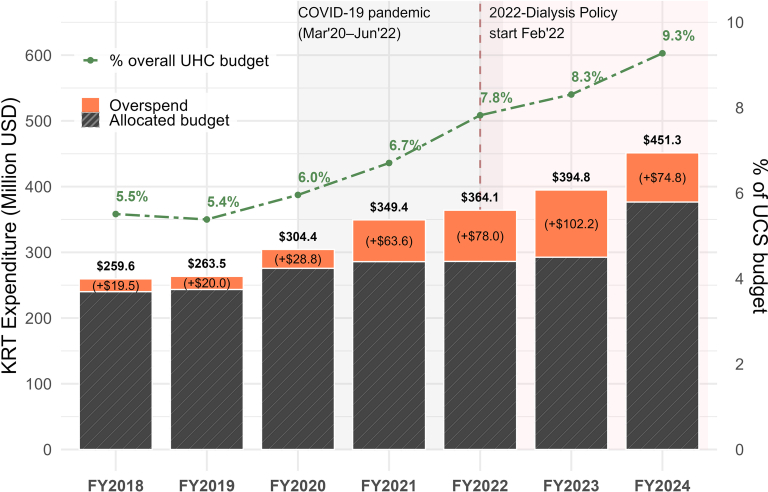
KRT expenditure and budget share, FY2018–FY2024. Stacked bars show total KRT expenditure (million USD), partitioned into allocated budget (striped) and overspend (orange). Values above bars indicate total expenditure; in-bar labels indicate overspend amounts. The dashed green line (right axis) shows KRT share of the UCS budget (%). The grey band denotes the COVID-19 period (March 2020–June 2022). The pink band and vertical dashed line mark dialysis policy implementation (February 2022), with partial exposure in FY2022. Fiscal years run from October to September. Expenditures include dialysis, vascular access, erythropoietin, peritoneal dialysis services, and kidney transplantation with immunosuppression, and exclude non-KRT care. Amounts are converted from Thai Baht using a fixed exchange rate (1 THB = 0.02945 USD). Data are summarized in Supplementary Table S2. FY, financial year; KRT, kidney replacement therapy; UCS, Universal Coverage Scheme; UHC, universal health coverage.

Expenditure on KRT consistently exceeded the allocated budget, resulting in a deficit that widened from THB 0.7 billion (USD 20 million) in 2018 to a peak of THB 3.5 billion (USD 102 million) in 2023, before narrowing to THB 2.5 billion (USD 75 million) in 2024. In absolute terms, KRT spending increased by 73.9% from 2018, reaching THB 15.0 billion (USD 452 million) in 2024. This accounted for 9.3% of the national Universal Health Coverage budget, up from 5.5% in 2018 (Figure 2, Supplementary Table S4).

### Changes in Patient Demographics and Dialysis Initiation Patterns

Among incident dialysis cases from January 2020 to September 2024, patient demographics and clinical characteristics remained broadly stable. Overall, the postpolicy cohort was slightly older and had a higher burden of comorbidities (Table 1). Modality-specific profiles—classified by intended dialysis modality ascertained at 90 days—showed minimal variation. Patients intended for HD maintained a consistent mean age and comorbidity index, whereas the PD group exhibited only marginal increases (Supplementary Table S5). Hypertension and diabetes mellitus remained the dominant comorbidities. Geographically, the Northeast region and urban areas remained the primary contributors (Table 1).

**Table 1:** Characteristics of patients initiating dialysis before and after the 2022 policy change

Demographic and clinical characteristics	Prepolicy (Jan 2020–Jan 2022) *n* = 26,467	Postpolicy (Feb 2022–Sep 2024) *n* = 55,089	*P*-value^a^
Age (yrs), mean ± SD	58.5 ± 13.8	60.1 ± 13.7	*< 0.001*
Age group, *n* (%)			*< 0.001*
< 20	280 (1.1%)	411 (0.7%)	
20–44	3561 (13.5%)	6602 (12.0%)	
45–59	8752 (33.1%)	16,628 (30.2%)	
60–74	11,290 (42.7%)	24,464 (44.4%)	
75+	2574 (9.7%)	6963 (12.6%)	
missing	10 (0.0%)	21 (0.0%)	
Male sex, *n* (%)	13,036 (49.3%)	28,179 (51.2%)	*< 0.001*
Missing	8 (0.0%)	21 (0.0%)	
Charlson Comorbidity Index, mean ± SD	6.0 ± 2.2	6.1 ± 2.2	
Charlson Comorbidity Index category, n (%)			*< 0.001*
0–2	1633 (6.2%)	3,236 (5.9%)	
3–4	4956 (18.7%)	9563 (17.4%)	
5–7	13,835 (52.3%)	28,237 (51.3%)	
≥8	6026 (22.8%)	14,011 (25.4%)	
missing	17 (0.1%)	42 (0.1%)	
Comorbidities (details)			
Hypertension, *n* (%)	25,105 (94.9%)	50,414 (91.6%)	< 0.001
Diabetes mellitus, *n* (%)	17,809 (67.3%)	36,744 (66.8%)	0.10
History of myocardial infarction, *n* (%)	2925 (11.1%)	5939 (10.8%)	0.25
Congestive heart failure, *n* (%)	8,898 (33.6%)	17,803 (32.3%)	< 0.001
Peripheral vascular disease, *n* (%)	855 (3.2%)	1914 (3.5%)	0.071
History of stroke/TIA, *n* (%)	3729 (14.1%)	8624 (15.7%)	< 0.001
Dementia, *n* (%)	111 (0.4%)	300 (0.5%)	0.018
Hemiplegia, *n* (%)	941 (3.6%)	2,215 (4.0%)	0.001
Liver disease (mild), *n* (%)	1878 (7.1%)	4240 (7.7%)	0.002
Liver disease (moderate to severe), *n* (%)	252 (1.0%)	507 (0.9%)	0.66
Chronic pulmonary disease, *n* (%)	2784 (10.5%)	5734 (10.4%)	0.64
Connective tissue disease, *n* (%)	794 (3.0%)	1583 (2.9%)	0.32
Peptic ulcer disease, *n* (%)	1460 (5.5%)	3319 (6.0%)	0.004
Any malignancy, *n* (%)	1133 (4.3%)	2632 (4.8%)	0.002
Metastatic solid tumor, *n* (%)	127 (0.5%)	339 (0.6%)	0.016
AIDs, *n* (%)	0 (0.00%)	0 (0.00%)	-
Registration and service characteristics			
Registered in urban area, n (%)	23,709 (89.58%)	49,701 (90.22%)	0.004
Region of registration, *n* (%)			< 0.001
Bangkok	2061 (7.8%)	5016 (9.1%)	
Central	4746 (17.9%)	8672 (15.7%)	
North	5087 (19.2%)	9598 (17.4%)	
East	2367 (8.9%)	5358 (9.7%)	
Northeast	9447 (35.7%)	21,331 (38.7%)	
South	2759 (10.4%)	5114 (9.3%)	
Dialysis initiation characteristics			
Unplanned dialysis initiation^b^*n* (%)	11,754 (44.4%)	33,127 (60.1%)	< 0.001
Initial dialysis modality, *n* (%)			
HD	15,830 (59.8%)	49,607 (90.0%)	< 0.001
PD	10,637 (40.2%)	5482 (10.0%)	
Main dialysis modality among alive patients at 3 mos^c^*n* (%)	(*n* = 24,126)	(*n* = 47,606)	
HD	8175 (33.9%)	40,354 (84.8%)	< 0.001
PD	15,951 (66.1%)	7252 (15.2%)	

HD, hemodialysis, PD, peritoneal dialysis; TIA, transient ischemic attack.

a *P*-values were derived from Pearson’s chi-squared test for categorical comparisons and from independent t-test for continuous measures.

b Unplanned dialysis initiation was defined as starting first treatment with HD with a nontunneled central venous catheter, expressed as a proportion of all incident dialysis patients.

c Main dialysis modality was defined as the treatment modality at 3 months among patients surviving ≥ 90 d after initiation.

Regarding initiation patterns, the proportion of patients starting on HD increased from 59.8% to 90.0%, whereas PD uptake at 90 days significantly decreased from 66.1% to 15.2% (Table 1). Consequently, the overall rate of unplanned dialysis initiation significantly increased from 44.4% to 60.1% (Table 1). When stratified by main dialysis modality (ascertained at day 90), unplanned initiation among HD patients increased from 58.0% to 65.3%, whereas rates among PD patients decreased sharply from 65.3% to 29.1% (Supplementary Table S5).

In the vascular access cohort, reliance on nontunneled central venous catheters at initiation increased in 2022 compared with the 2020–2021 baseline, accompanied by prolonged catheter duration. In subsequent years (2023–2024), these patterns improved, with higher arteriovenous fistula or graft use at initiation, increased conversion to permanent access, and shorter catheter duration (Supplementary Table S6).

### Impact of the 2022 Policy on Patient Survival

**Figure 3:**
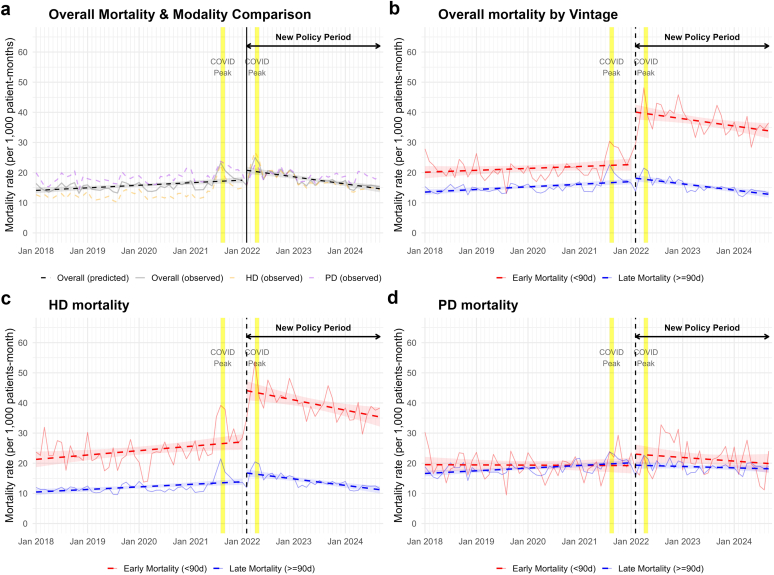
Mortality trends before and after the 2022 dialysis policy. Monthly mortality rates (deaths/1000 patient-mos) are shown for (a) overall dialysis, HD, and PD; (b) overall mortality by vintage (< 90 vs. ≥ 90 days); (c) HD mortality by vintage; and (d) PD mortality by vintage. Solid lines indicate observed rates; dashed lines indicate fitted trends from segmented regression models with 95% confidence intervals (shaded). Yellow bands denote COVID-19 mortality peaks. The vertical dashed line marks policy implementation (February 2022). In panel (a), orange and purple represent HD and PD, respectively; in panels (b–d), red and blue represent early (< 90 days) and late (≥ 90 days) mortality. HD, hemodialysis; PD, peritoneal dialysis.

At the system level, the 2022 policy implementation was associated with a significant increase in overall mortality risk (IRR: 1.19, 95% CI: 1.09–1.30) (Figure 3a and Supplementary Table S7). This elevation was predominantly driven by early mortality (< 90 days), which exhibited a substantial 77% immediate increase (IRR: 1.77, 95% CI: 1.59–1.97), whereas overall late mortality (≥ 90 days) did not show a significant immediate level change (IRR: 1.07, 95% CI: 0.98–1.16). HD mortality was significantly increased in all vintage groups, especially early mortality. In contrast, PD—regarding both early and late mortality—did not experience a significant immediate change. Following this immediate impact, the overall system mortality risk demonstrated a significant monthly decline during the postimplementation period (IRR: 0.985, 95% CI: 0.981–0.989), indicating a subsequent stabilization of the trend (Figure 3 and Supplementary Table S7).

At the individual level, survival in the incident cohort declined markedly in the postpolicy period. The mortality rate increased from 243.3 to 338.9 per 1000 patient-years, whereas median survival shortened from 3.2 to 2.3 years (Table 2). In multivariable analysis, the postpolicy period was independently associated with increased all-cause mortality (adjusted hazard ratio [aHR]: 1.40, 95% CI: 1.37–1.43). This risk remained elevated for both early (<90 days; aHR: 1.38, 95% CI: 1.32–1.45) and late (^3^90 days; aHR: 1.40, 95% CI: 1.36–1.44) mortality (Table 2 and Supplementary Table S8). Notably, the composition of mortality shifted significantly: early deaths comprised 36.6% of total deaths in the postpolicy period, compared with only 14.4% in the prepolicy period (Table 2). In a sensitivity analysis restricted to established HD patients (the vascular access cohort), the use of permanent vascular access by day 90 was identified as a strong protective factor (aHR: 0.11, 95% CI: 0.11–0.12). After adjusting for vascular access use, the policy itself was no longer significantly associated with early mortality (aHR: 1.05, *P* = 0.09); however, the risk for late mortality remained significantly elevated (aHR: 2.01, 95% CI: 1.91–2.11) (Supplementary Table S9).

**Table 2:** Survival outcomes among the incident dialysis cohort before and after the 2022 policy

Mortality type and summary statistics	Pre-policy (Jan 2020–Jan 2022) *n* = 26,467	Post-policy (Feb 2022–Sep 2024) *n* = 55,089	Adjusted HR^a^ for postpolicy vs. prepolicy (95% CI)
Overall mortality			1.40 (1.37–1.43), *P* < 0.001
Mortality rate (/1000 patient-yrs)	243.3	338.9	
Median survival^b^	3.2 yrs	2.3 yrs	
Early mortality (< 90 d)^c^			1.38 (1.32–1.45), *P* < 0.001
Number of deaths, *n* (%)	2297 (8.7%)	7391 (13.4%)	
%share to overall mortality	14.4%	36.6%	
Late mortality (≥ 90 d)^d^			1.40 (1.36–1.44), *P* < 0.001
Mortality rate (/1000 patient yrs)	229.8	267.8	
Median survival^b^	3.3 yrs	2.3 yrs	

95% CI, 95% confident interval; HR, hazard ratio;.

a Adjusted hazard ratio for postpolicy vs. prepolicy from a multivariable Cox model measured from dialysis initiation, adjusted for unplanned initiation, age, sex, Charlson category, and region.

b Median survival in the overall row is measured from dialysis initiation (day 0), whereas median survival in the late mortality row is conditional on surviving the first 90 d.

c Early mortality was defined as death within 90 days of dialysis initiation. Follow-up was truncated at 90 d, so early mortality rates reflect short-term hazards and should not be interpreted as annualized rates.

d Late mortality was defined as death occurring ≥ 90 d after dialysis initiation. Analyses used a 90-d landmark: only patients alive at day 90 entered the risk set, and survival time was measured from that point onward.

## Discussion

In February 2022, a new KRT policy emphasizing patient choice was introduced to expand treatment options and address perceived inequities inherent in the long-standing PD-first approach. Although well-intentioned, our nationwide analysis revealed significant unintended consequences, including rapid growth in overall dialysis utilization, a precipitous decline in PD, escalating expenditures, and increased early mortality among incident patients. Regarding KT, although the absolute volume increased, driven by a concurrent national project commemorating the 100th Anniversary of Her Royal Highness Princess Galyani Vadhana^20^— the rapid expansion of the dialysis population meant that the individual probability of receiving a transplant remained stagnant.

These findings raise critical concerns regarding sustainability, because KRT already consumes nearly 10% of the health budget to serve <1% of the population. To place these costs in context, direct dialysis expenditure reached USD 452.1 million in FY2024, equivalent to approximately 0.09% of Thailand’s GDP (USD ∼526.4 billion), in a middle-income country with a GDP per capita of USD 7350.^21^ At the country income–level, such expenditure represented a substantial opportunity cost for the health system, given that the per-capita public health spending is approximately USD 200–220/person/yr, constraining fiscal space for other essential health services. Moreover, this figure (USD ∼ 452 million) likely underestimated the true financial burden, because it included only costs directly attributable to KRT and excluded expenditures related to comorbidity management, nonerythropoietin medications, and inpatient or outpatient care for dialysis-related complications.

Furthermore, declining PD volumes threaten economies of scale in procurement and risk the progressive deskilling of nephrologists and PD nurses,^11^^,^^22^ whereas the surging HD demand has outpaced workforce capacity, driving rapid private-sector expansion—facilitated by the withdrawal of accreditation requirements and commercial opportunities—without commensurate staffing levels.^16^ Observed consequences include increased reliance on temporary catheters, and higher early mortality, which prompted the NHSO to urgently commission corrective reforms.^17^

Adopting a patient-choice model aligns with global best practices, offering potential for improved quality of life and preference-concordant care.^23^, ^24^, ^25^ This is particularly relevant in Thailand, where caregiver shortages and the burden of home-based PD often drive families toward HD.^26^, ^27^, ^28^ Local data indicate that > 50% of Thai PD patients require family assistance,^27^ whereas a substantial proportion of patients in rural areas preferred to decline dialysis specifically to avoid burdening their families.^26^ Crucially, however, realizing these benefits requires timely referral, comprehensive predialysis education, and effective shared decision-making,^29^^,^^30^ supported by adequate infrastructure, such as accessible HD centers and trained personnel.^31^

In practice, our findings suggest that system-level realities constrained these theoretical goals. The rapid HD expansion was largely driven by supply-side incentives following the increased role of private-sector providers and the absence of effective gatekeeping mechanisms, such as preauthorization. Informal financial arrangements emerged where referring providers reportedly received referral fees of THB 150–250 (USD 4–7) per session for directing patients to private HD centres.^22^ These incentives alongside persistent misconceptions regarding HD superiority,^22^^,^^29^^,^^32^ likely biased counselling and distorted treatment selection favoring HD. Consequently, these dynamics and suboptimal shared decision-making may have marginalized nondialytic options, contributing to the initiation of dialysis in frail elderly patients for whom conservative care might have been appropriate.^33^

Regarding the increased postpolicy mortality, although aggregate characteristics appeared broadly similar, this likely reflected the averaging of heterogeneous pathways rather than true clinical equivalence. Although specific cause-of-death data were unavailable, we hypothesize that the postpolicy cohort comprised high-risk subgroups obscured by summary measures: (i) frail older patients initiating HD with limited survival benefit, (ii) patients initiating dialysis earlier without strict clinical indication, and (iii) patients with acute kidney injury—a group with high short-term mortality^35^—potentially misclassified as kidney failure due to reimbursement incentives. Furthermore, system-level pressures during rapid expansion—particularly following the relaxation of accreditation standards—likely contributed, alongside reports of shortened dialysis sessions and staffing constraints.^22^ Our sensitivity analysis further implicates temporary catheters as a primary driver: adjusting for vascular access nullified the policy’s association with early death.^34^ This spike attenuated over time alongside improved access planning, suggesting system adaptation.

Crucially, the increased mortality trends likely do not solely reflect a selection effect involving patients who would have been excluded prior to the policy change. The antecedent “PD First” policy was not a “no dialysis” mandate, because patients could still receive HD when clinically indicated. Moreover, the concentration of excess mortality within the first 90 days challenges the interpretation that this was simply a consequence of expanded access for high-risk groups. However, definitive assessment was limited by the absence of a national pr-dialysis chronic kidney disease registry capturing untreated disease trajectories, underscoring the need for cautious interpretation and continued follow-up studies to evaluate longer-term outcomes.

Several limitations warrant consideration. First, the reliance on administrative data precluded analysis of cause-of-death and granular clinical variables (e.g., frailty, laboratory values, timing of dialysis initiation, and residual kidney function). Second, the absence of catheter-to-dialysis intervals prevented distinguishing unplanned PD, likely underestimating its prevalence. Third, potential miscoding of acute kidney injury may have inflated early mortality. Finally, restriction to the UCS population limits generalizability across schemes and long-term evaluation, and residual confounding cannot be excluded. Despite these limitations, the findings remain highly informative for understanding the system-level effects of the policy change.

This experience highlights that policy reforms in KRT require meticulous preparation, stakeholder engagement, and investment in system readiness to achieve their aims without compromising quality or sustainability. In contrast to Thailand’s original PD-first policy in 2008, which was developed with extensive planning, infrastructure investment, and workforce training.^11^^,^^17^ The 2022 reform was introduced rapidly and, despite good intentions, was followed by unintended consequences. This study, together with other studies conducted as part of the Commission,^17^ provided insights into and recommendations to the NHSO to inform policy improvement. In response, the NHSO has proactively adopted corrective measures initiated in April 2025, including reinstating a PD-preferred approach, implementing preauthorization for dialysis initiation, formalizing triage for conservative care suitability, and strengthening shared decision-making.^17^ A multistakeholder working group has been established to oversee implementation, monitoring, and evaluation.

In conclusion, Thailand’s 2022 dialysis policy reform has resulted in rapid expansion of services, rising expenditure, and increased early mortality. Safeguards are required to preserve PD capacity, strengthen shared decision-making and conservative kidney management, realign incentives, and reinforce quality monitoring. For other low- and middle-income countries, Thailand’s experience underscores the importance of pairing coverage expansion with the preservation of home-therapy capacity, robust predialysis pathways, and effective governance.

## Disclosure

JP has received speaking honoraria from Vantive Healthcare and currently receives a fellowship grant from the UK International Science Partnership Fund. TK has received consultancy fees from Visterra, Otsuka, and AstraZeneca as a country investigator; and is currently a recipient of a research grant from the National Research Council of Thailand. TK has also received speaking honoraria from AstraZeneca, Alexion, Otsuka, Fresenius Medical Care, and Baxter Healthcare. All the other authors declared no competing interests.

## Supplementary Material

Supplementary File (PDF) **Supplementary Methods. Figure S1.** Impact of the 2022 policy on absolute patient volumes. **Figure S2.** Temporal trends in dialysis patient distribution by provider ownership type. **Table S1.** Summary of data sources for each analysis in this study. **Table S2.** ICD-10 codes used to define Charlson comorbidity categories. **Table S3.** Impact of the 2022 policy on dialysis service utilization, modality shifts, and kidney transplantation. **Table S4.** Kidney replacement therapy expenditure and budget share, FY2018–FY2024. **Table S5.** Baseline characteristics of incident dialysis patients by policy period and main dialysis modality. **Table S6.** Trends in initial vascular access and conversion to permanent access among incident HD patients, 2020–2024. **Table S7.** Interrupted time series analysis of dialysis mortality before and after the 2022 policy. **Table S8.** Multivariable Cox models for mortality among incident dialysis patients. **Table S9.** Sensitivity analysis of multivariable Cox models for mortality among incident HD patients vascular access cohort (2020–2024) considering conversion to permanent vascular access use by 90-days. Strengthening the reporting of observational studies in epidemiology (STROBE) checklist.

Supplementary File (xlsx) **Monthly aggregated data supplements (xlsx)****.**

## Footnotes

Supplementary File (PDF and xlsx)

**Supplementary Methods.**

**Monthly aggregated data supplements (xlsx).**
**Figure S1.** Impact of the 2022 policy on absolute patient volumes. **Figure S2.** Temporal trends in dialysis patient distribution by provider ownership type. **Table S1.** Summary of data sources for each analysis in this study. **Table S2.** ICD-10 codes used to define Charlson comorbidity categories. **Table S3.** Impact of the 2022 policy on dialysis service utilization, modality shifts, and kidney transplantation. **Table S4.** Kidney replacement therapy expenditure and budget share, FY2018–FY2024. **Table S5.** Baseline characteristics of incident dialysis patients by policy period and main dialysis modality. **Table S6.** Trends in initial vascular access and conversion to permanent access among incident HD patients, 2020–2024. **Table S7.** Interrupted time series analysis of dialysis mortality before and after the 2022 policy. **Table S8.** Multivariable Cox models for mortality among incident dialysis patients. **Table S9.** Sensitivity analysis of multivariable Cox models for mortality among incident HD patients vascular access cohort (2020–2024) considering conversion to permanent vascular access use by 90-days. Strengthening the reporting of observational studies in epidemiology (STROBE) checklist.

## References

[1] Flythe J.E., Watnick S. (2024). Dialysis for chronic kidney failure: a review. JAMA.

[2] Tonelli M., Wiebe N., Knoll G. (2011). Systematic review: kidney transplantation compared with dialysis in clinically relevant outcomes. Am J Transplant.

[3] Nyokabi P., Youngkong S., Bagepally B.S. (2024). A systematic review and quality assessment of economic evaluations of kidney replacement therapies in end-stage kidney disease. Sci Rep.

[4] Larpparisuth N., Cheungpasitporn W., Lumpaopong A. (2021). Global perspective on kidney transplantation: Thailand. Kidney360.

[5] Sangthawan P., Klyprayong P., Geater S.L. (2022). The hidden financial catastrophe of chronic kidney disease under universal coverage and Thai “peritoneal dialysis First Policy”. Front Public Health.

[6] Luyckx V.A., Miljeteig I., Ejigu A.M., Moosa M.R. (2017). Ethical challenges in the provision of dialysis in resource-constrained environments. Semin Nephrol.

[7] Sirivongs D., Kasemsup V., Wansiripaisal A., Janma J. (2011).

[8] Tangcharoensathien V., Kasemsup V., Teerawattananon Y., Supaporn T., Vasavid C., Prakongsai P. (2005).

[9] Tangcharoensathien V., Pitayarangsarit S., Patcharanarumol W. (2013). Promoting universal financial protection: how the Thai universal coverage scheme was designed to ensure equity. Health Res Policy Syst.

[10] Teerawattananon Y., Mugford M., Tangcharoensathien V. (2007). Economic evaluation of palliative management versus peritoneal dialysis and hemodialysis for end-stage renal disease: evidence for coverage decisions in Thailand. Value Health.

[11] Chuengsaman P., Kasemsup V. (2017). PD First policy: Thailand’s response to the challenge of meeting the needs of patients with end-stage renal disease. Semin Nephrol.

[12] MGR Online (2011). The Kidney Disease Patients' Association protests against unequal access to dialysis under the universal healthcare scheme. https://mgronline.com/qol/detail/9540000061292.

[13] MGR Online (2024). PD first policy “Violation of rights” or “Patients have access to treatment” 2016 8 March. https://mgronline.com/qol/detail/9590000095013.

[14] National Health Security Office (2022). New HD cost reimbursement policy will ease heavy burden on kidney patients. https://eng.nhso.go.th/view/1/DescriptionNews/New-HD-cost-reimbursement-policy-will-ease-heavy-burden-on-kidney-patients/416/EN-US#:%7E:text=The%20decision%20by%20the%20National,all%20kidney%20patients%2C%20he%20said.

[15] Botwright S., Teerawattananon Y., Phannajit J. (2025). Balancing patient choice and health system capacity: a system dynamics model of dialysis in Thailand. BMC Med.

[16] Satirapoj B., Tantiyavarong P., Chuasuwan A. (2025). Thailand renal replacement therapy registry 2023 annual data report: dialysis center providers in Thailand. J Nephrol Soc Thai.

[17] Teerawattananon Y., Chavarina K.K., Phannajit J. (2026). Nature Medicine Commission on dialysis policy in low- and middle-income countries. Nat Med.

[18] Bernal J.L., Cummins S., Gasparrini A. (2016). Interrupted time series regression for the evaluation of public health interventions: a tutorial. Int J Epidemiol.

[19] Exchange rates UK (2024). Thai Baht to U S Dollar History. https://www.exchangerates.org.uk/THB-USD-spot-exchange-rates-history-2024.html.

[20] Chanchairujira T., Kanjanabuch T., Pongskul C., Sumethkul V., Supaporn T. (2023). Dialysis and kidney transplant practices and challenges in Thailand. Nephrology (Carlton).

[21] The World Bank (2024). GDP (current US$). https://data.worldbank.org/indicator/NY.GDP.MKTP.CD?locations=TH.

[22] Botwright S., Teerawattananon Y., Yongphiphatwong N. (2025). Understanding healthcare demand and supply through causal loop diagrams and system archetypes: policy implications for kidney replacement therapy in Thailand. BMC Med.

[23] Kidney Disease: Improving Global Outcomes (KDIGO) Acute Kidney Injury Work Group (2024). KDIGO 2024 Clinical practice guideline for the evaluation and management of chronic kidney disease. Kidney Int.

[24] Park B.H., Shin H.S., Kim J. (2024). Effects of shared decision-making on the prognosis of peritoneal dialysis patients. Medicine (Baltimore).

[25] Kazi B.S., Duberstein P.R., Kluger B.M. (2023). Prevalence and correlates of preference-concordant care among hospitalized people receiving maintenance dialysis. Kidney360.

[26] Hengtrakulvenit J., Prommachat K., Promnim S. (2021). A retrospective comparison of Thai patients with end-stage renal disease who chose to decline or receive dialysis therapy. J Dep Med Serv.

[27] Puapatanakul P., Kanjanabuch T., Tungsanga K. (2022). Assisted peritoneal dialysis performed by caregivers and its association with patient outcomes. Perit Dial Int.

[28] Parapiboon W., Pitsawong W., Wongluechai L., Thammavaranucupt K., Raegasint L. (2020). Customized versus conventional video counseling for peritoneal dialysis decision-making in patients with stage 5 chronic kidney disease under a PD-first policy: a randomized controlled study. Kidney Res Clin Pract.

[29] Yongphiphatwong N., Teerawattananon Y., Supapol P. (2025). The way home: a scoping review of public health interventions to increase the utilization of home dialysis in chronic kidney disease patients. BMC Nephrol.

[30] Machowska A., Alscher M.D., Reddy Vanga S. (2016). Factors influencing access to education, decision making, and receipt of preferred dialysis modality in unplanned dialysis start patients. Patient Prefer Adherence.

[31] Iyengar A., Kalyesubula R., Darwish R., Luyckx V.A. (2025). International equity in access to home dialysis. Curr Opin Nephrol Hypertens.

[32] Tungsanga K., Kanjanabuch T., Mahatanan N., Praditpornsilp K., Avihingsanon Y., Eiam-Ong S. (2008). The status of, and obstacles to, continuous ambulatory peritoneal dialysis in Thailand. Perit Dial Int.

[33] Davison S.N., Pommer W., Brown M.A. (2024). Conservative kidney management and kidney supportive care: core components of integrated care for people with kidney failure. Kidney Int.

[34] Phannajit J., Kanjanabuch T., Ponpirul K. (2021). Unplanned peritoneal dialysis (PD) and patient survival under Thailand “PD first” policy. Nephrology.

[35] Kellum J.A., Romagnani P., Ashuntantang G., Ronco C., Zarbock A., Anders H.-J. (2021). Acute kidney injury. Nat Rev Dis Primers.

