# Supplementary material for: Outcomes and Trade-offs of Thailand’s 2022 Patient-Choice Dialysis Policy Reform
Source: Kidney Int Rep. 2026 Feb 25;11(5):106377. doi: 10.1016/j.ekir.2026.106377 (PMC13087778; doi:10.1016/j.ekir.2026.106377)
Supplement: Supplementary File (PDF) — Supplementary Methods. Figure S1. Impact of the 2022 policy on absolute patient volumes. Figure S2. Temporal trends in dialysis patient distribution by provider ownership type. Table S1. Summary of data sources for each analysis in this study. Table S2. ICD-10 codes used to define Charlson comorbidity categories. Table S3. Impact of the 2022 policy on dialysis service utilization, modality shifts, and kidney transplantation. Table S4. Kidney replacement therapy expenditure and budget share, FY2018–FY2024. Table S5. Baseline characteristics of incident dialysis patients by policy period and main dialysis modality. Table S6. Trends in initial vascular access and conversion to permanent access among incident HD patients, 2020–2024. Table S7. Interrupted time series analysis of dialysis mortality before and after the 2022 policy. Table S8. Multivariable Cox models for mortality among incident dialysis patients. Table S9. Sensitivity analysis of multivariable Cox models for mortality among incident HD patients vascular access cohort (2020–2024) considering conversion to permanent vascular access use by 90-days. Strengthening the reporting of observational studies in epidemiology (STROBE) checklist. [file mmc1.pdf]

**Supplementary materials** *for Phannajit J, et al. Outcomes and Trade-offs of Thailand's 2022 Patient-Choice Dialysis Policy Reform*

Table of Contents

Supplementary Methods ........ 2

1. Data sources and linkage ........ 2

Supplementary Table S1. Summary of data sources for each analysis in this study ........ 3

2.Definition of study periods ........ 4

3.Study populations ........ 4

4.Measures and operational definitions ........ 4

Supplementary Table S2: ICD-10 codes used to define Charlson comorbidity categories ........ 6

5.Analytical methods........ 7

6. Use of language models ........ 13

Supplementary Figure S1. Impact of the 2022 Policy on Absolute Patient Volumes. ........ 14

Supplementary Figure S2. Temporal trends in dialysis patient distribution by provider ownership type ........ 15

Supplementary Table S3. Impact of the 2022 Policy on Dialysis Service Utilisation, Modality Shifts, and Kidney Transplantation ........ 16

Supplementary Table S4. Kidney replacement therapy (KRT) expenditure and budget share, FY2018–FY2024. ........ 17

Supplementary Table S5. Baseline characteristics of incident dialysis patients by policy period and main dialysis modality ........ 18

Supplementary Table S6. Trends in initial vascular access and conversion to permanent access among incident HD patients, 2020–2024. ........ 20

Supplementary Table S7. Interrupted time series (ITS) analysis of dialysis mortality before and after the 2022 policy........ 21

Supplementary Table S8. Multivariable Cox models for mortality among incident dialysis patients ........ 22

Supplementary Table S9. Sensitivity analysis of multivariable Cox models for mortality among incident HD patients considering conversion to permanent vascular access use by 90-day........ 23

Strengthening the reporting of observational studies in epidemiology (STROBE) checklist ........ 24

# Supplementary Methods

## 1. Data sources and linkage

The primary datasets were the Chronic Kidney Disease–Disease Management Information System (CKD-DMIS; established 2008, available through 2024) and the National Health Security Office (NHSO) electronic claims database (e-Claim) available from 2016 through 2024). CKD-DMIS contains near real-time records for patients receiving kidney replacement therapy (KRT) under universal coverage, including registration, modality history, peritoneal dialysis (PD) supply claims, and detailed hemodialysis (HD) session data. The e-Claim database provides demographics, ICD-10 diagnoses, ICD-9 procedures, inpatient and outpatient encounters, and reimbursed costs. Mortality was ascertained via linkage to the Central Civil Registration database (Ministry of Interior). Provider type and ownership were classified using the NHSO provider registry, cross-checked against the Thailand Renal Replacement Therapy Registry (TRT) available from 2008 onwards). Budget and expenditure data for fiscal years 2018–2024 were extracted from NHSO finance reports.

Datasets were linked deterministically using an encrypted unique identification number. Quality checks included de-duplication of person records; logical validation of initiation dates (e.g., first HD/PD claim precedes follow-up sessions; PD solution claims align with recorded starts); internal consistency of modality timelines; and cross-validation of deaths between CKD-DMIS and Civil Registration.

Information on allocated budgets and expenditures for KRT including HD, PD and kidney transplantation was provided by the NHSO for fiscal years 2018–2024. In Thailand, the fiscal year runs October–September (e.g., FY2024 = October 2023–September 2024).

For all analyses, the analytic period was restricted to January 2018–September 2024, although database inception years are shown in Supplementary Method Table M1.

Supplementary Table S1. Summary of data sources for each analysis in this study

| Type of data | Key variables | Level of record | Source | Owner | Database availability | Analysis period |
|---|---|---|---|---|---|---|
| Kidney replacement therapy related data | Registration date & site; date of initiation; modality history; vascular access; HD session details (prescription, duration, access, provider); kidney transplantation | Patient-level / per registration / dialysis sessions | CKD–Disease Management Information System (CKD-DMIS) | National Health Security Office (NHSO) | 2008-present | Jan 2018 – Sep 2024 |
| Comorbidities | ICD-10 diagnoses; ICD-9 procedures; inpatient/outpatient encounters; reimbursed costs | Patient-level per visit | Electronic claim database (e-claim) | NHSO | 2016-present | All linked data (last accessed 1 Sep 2025) |
| Deaths | Vital status; date of death | Patient-level | Central Civil Registration | NHSO | NA | All linked data (Since inception to 1 Sept 2024) |
| Registered hospital / dialysis facility | Region; facility type/level; ownership; location | Center-level | NHSO's provider registry | NHSO | 2018-2024 | Jan 2018-Sept 2024 |
| | Presence of HD facility; ownership (in-house vs outsourced) | Center-level | Thailand's dialysis registry | Nephrology Society of Thailand | 2008-2024 | Jan 2018-Sept 2024 |
| Budget & expenditures | Allocated budget and executed expenditures by fiscal year; line items for KRT activities (e.g., PD supplies, HD sessions, access procedures), where available | Fiscal year (FY); | NHSO budget & finance reports | NHSO | FY2018–FY2024 | FY2018–FY2024; Fiscal year runs Oct–Sep (e.g., FY2024 = Oct 2023–Sep 2024) |

Note: NA: Not applicable; Fiscal year in Thailand starts on 1 October of each year.

## 2.Definition of study periods

The study period covered 1 January 2018 to 1 September 2024 (81 months in total). The **pre-policy period** was defined as 1 January 2018 to 31 January 2022 (49 months), and the **post-policy period** as 1 February 2022 to 1 September 2024 (32 months), corresponding to the implementation of the new dialysis policy.

## 3.Study populations

We analysed three complementary populations:

1. **System-level analyses (prevalent dialysis population).**
   Including all UCS patients alive on maintenance dialysis on the first day of each month from January 2018 to September 2024, used to construct monthly denominators for prevalence and mortality trends.

2. **Incident dialysis cohort**
   Including all patients initiating maintenance HD or PD between 1 January 2020 and 1 September 2024. This restricted window was selected to ensure complete ascertainment of baseline characteristics and treatment data. This cohort was used to describe patient demographics, initial modality choice, modality status at day 90, and survival outcomes. Modality at death was attributed using the most recent modality, provided it had been in place for at least 14 days prior to death, to reduce bias from terminal transfers. Patients switching to PD or kidney transplantation within 90 days were excluded from vascular access analyses, but patients who died within 90 days were retained.

3. **Vascular access cohort**
   A subset of HD initiators from the same period (1 January 2020 to 1 September 2024, when systematic recording of vascular access type became mandatory), analysed for access type and conversion rates, as systematic recording of vascular access data commenced in 2020. Patients who switched to PD or received a transplant within 90 days were excluded, while those who died within 90 days were retained.

## 4.Measures and operational definitions

*End-stage kidney disease*

Patients were classified as having ESKD if registered in CKD-DMIS with evidence of maintenance dialysis initiation (HD or PD) or kidney transplantation during the analytic period. CKD-DMIS is designed exclusively for chronic KRT under the UCS and does not capture acute kidney injury (AKI) treatments. During the study period,

there was no pre-authorisation requirement to confirm ESKD status at initiation; therefore, initiation was validated by service claims (first HD session, first PD session record, or first PD fluid claim).

*Incident cases*

Defined as patients with a first record of dialysis or kidney transplantation during the study period, with no prior history of dialysis (>3 months) or transplantation.

*Unplanned dialysis initiation*

Defined as as initiation of KRT with HD using a non-tunnelled central venous catheter. This definition includes patients who ultimately received PD but required temporary HD at initiation.

*Initial dialysis modality*

The modality with which a patient first initiated treatment, classified as HD or PD.

*Main modality at 90 days*

Defined among patients alive and without transplantation at day 90. Patients who switched back within 90 days were classified according to their eventual stabilised modality. For patients who died within 90 days, the last recorded modality was retained.

*Modality at death*

To reduce bias from terminal transfers, deaths were attributed to the chronic modality if it had been in place for ≥14 days prior to death; otherwise, they were attributed to the most recent modality.

*Modality shift*

Defined as a switch from HD to PD, or vice versa, without return to the original modality within 90 days or receipt of kidney transplantation.

*Charlson comorbidity index (CCI)*

Derived from ICD-10 codes (Supplementary method table M2) in NHSO claims using the Quan algorithm[1].. The CCI was analysed categorically (0–2, 3–4, 5–7, ≥8), and individual comorbidities were reported separately.

Supplementary Table S2: ICD-10 codes used to define Charlson comorbidity categories

| Charlson category | ICD-10 codes included |
|---|---|
| Myocardial infarction | I21-I22, I252 |
| Congestive heart failure | I099,I110,I255,I130,I132,I42,I43,I50,P290 |
| Peripheral vascular disease | I70,I71,I731,I738,I739,I771.I790,I792,K551,K558,K559,Z958,Z959 |
| Cerebrovascular disease | G45,G46,I60-I69,H340 |
| Dementia | F01-F03,F051,G30,G311 |
| Hemiplegia/paraplegia | G041,G114,G801,G802,G81,G82,G830-4,G839 |
| Chronic lung disease | I278,I279,J40-7,J60-7,J684,J701,J703 |
| Rheumatologic disease | M05,M06,M315,M32,M33,M34,M351,M353,M360 |
| Peptic ulcer disease | K25-28 |
| Diabetes mellitus* | E10-14 |
| Mild liver disease | B18,K700-K703. K709,K713-K715,K717,K73,K74,K760,K762-K764,K768,K769.Z944 |
| Moderate/severe liver disease | I850,I856,I864,I982,K704,K711,K721,K729,K765-K767 |
| Any malignancy | C00-C26,C30-C34.C37-C41.C43,C45-C58,C60-C76,C81-C85,C88,C90-C97 |
| Metastatic solid tumour | C77-C80 |
| AIDS | B20-B22 (AIDS) excluding B24 (asymptomatic HIV) alone |

*All patients in this study considered as diabetes with complications due to end-stage kidney disease

Abbreviations: HIV = Human immunodeficiency virus, AIDS = Acquired immunodeficiency syndrome

## 5.Analytical methods

### *5.1 Analysis and data manipulation software*

Data were extracted and transformed using Apache Hive SQL (Apache Software Foundation) and queried with DBeaver Community Edition 25.2.0 (DBeaver Corp). Statistical analyses were conducted in Stata/SE 19.5 (StataCorp, College Station, TX). Figures were produced primarily with ggplot2 in R 4.4.2 (R Foundation for Statistical Computing, Vienna, Austria), with additional visualisations created in Stata.

Analyses used two-sided tests with $p<0.05$; effect sizes are reported with 95% confidence intervals; no adjustments were made for multiple comparisons and results should be regarded as hypothesis-generating.

### *5.2 Prevalence and incidence measures*

Monthly prevalence was defined as the number of active dialysis patients in a given month divided by the UCS population for that month, expressed per 1,000,000 population.

Monthly incidence was defined as the number of new dialysis starts in a given month divided by the UCS population, expressed per 1,000,000 population.

Modality shifts were defined as the number of patients switching from HD to PD, or vice versa, divided by the number of patients on the originating modality, expressed per 1,000 patients.

Kidney transplantation (KT) incidence was defined as the number of new KT procedures per month per 1,000 active dialysis patients.

### *5.3 Interrupted time-series (ITS) analysis*

To evaluate the impact of the 2022 "HD First" policy implementation on dialysis service provision and patient outcomes, we employed a single-group Interrupted Time Series (ITS) design. This quasi-experimental approach allows for the assessment of longitudinal effects by comparing trends before and after a defined intervention point, whilst accounting for pre-existing secular trends.

Model Specification: To account for over-dispersed count data, we fitted **Generalized Linear Models (GLM) with a negative binomial distribution** and a logarithmic link function. The regression equation was specified as:

$$\ln(Y_t) = \beta_0 + \beta_1 \cdot time_t + \beta_2 \cdot policy_t + \beta_3 \cdot time\_after_t + \ln(exposure_t) + \varepsilon$$

Where:

- $Y_t$: The outcome count at month $t$.
- $time_t$: A continuous variable indicating months elapsed from the start of the study (January 2018), capturing the pre-policy **baseline trend** ($\beta_1$).
- $policy_t$: A binary indicator (0 prior to February 2022; 1 thereafter) capturing the **immediate level change** ($\beta_2$) at implementation.
- $time\_after_t$: A continuous variable indicating months elapsed since policy implementation (coded 0 prior to February 2022), capturing the **post-policy slope change** ($\beta_3$).
- $exposure_t$: The natural logarithm of the population denominator, included to estimate rates.

Outcome Measures and Offsets:

Exponentiated coefficients are reported as Incidence Rate Ratios (IRR). To estimate rates correctly, specific denominators were included as exposure offsets:

- **Dialysis Prevalence & Incidence**: The mid-year Universal Coverage Scheme (UCS) population was used as the denominator.
- **Modality Shifts, Transplantation & Mortality:** The total prevalent (active) dialysis population served as the denominator.

Mortality and Vintage Definitions:

Mortality analyses were stratified by modality and vintage, with specific rules applied to reduce bias:

- **Modality Attribution:** To minimise bias from terminal transfers, deaths were attributed to the chronic modality if it had been maintained for $\ge 14$ days prior to the event; otherwise, the death was attributed to the immediately preceding modality.
- **Vintage**: Stratified into **early mortality** (occurring within 90 days of initiation) and **late mortality** (occurring at or beyond 90 days).

Model assumptions were verified through post-estimation diagnostics:

- Overdispersion: Validated using the Likelihood Ratio test of the dispersion parameter, which confirmed the necessity of the negative binomial distribution across all primary models ($p < 0.001$).

- Serial Correlation: Assessed by inspecting Anscombe residuals for non-random patterns and confirmed via the Portmanteau ($Q$) statistic.
- Linearity & Fit: Evaluated by plotting deviance residuals against time with Lowess smoothing to ensure model adequacy.
- Contextual Factors: National COVID-19 mortality peaks (August 2021 and April 2022) were identified for temporal context but were not statistically adjusted for in the models.

*5.4 Distribution of dialysis providers*

We examined the distribution of HD and PD patients across provider types on a monthly basis from January 2018 to September 2024.

- **HD**: Providers were categorised as:
    1. Publicly owned HD centres
    2. Public hospitals with outsourced HD provided by private contractors
    3. Private hospitals or stand-alone clinics

If a facility operated both in-house and outsourced HD, it was classified as **outsourced**.

- **PD**: Providers were classified as public or private (outsourcing does not occur in PD).

For each month, proportions were calculated as the number of patients in each provider type divided by the total active patients on that modality. Average proportions were summarised for the pre-policy (Jan 2018–Jan 2022) and post-policy (Feb 2022–Sep 2024) periods.

*5.5 Kidney replacement therapy (KRT) expenditures*

We summarized program financing using NHSO budget and finance reports. For each fiscal year (FY2018–FY2024), each fiscal year runs from 1$^{st}$ October of the previous year to 30$^{th}$ September of the fiscal year (e.g., FY2024 = Oct 2023–Sep 2024). The following measures were reported:

- **National universal health-care budget** (A) — the annual Universal Coverage Scheme (UCS) allocation.
- **Allocated budget to KRT services** (B) — the annual line-item allocation earmarked for KRT.
- **Actual expenditure on KRT** (C) — realized spending for KRT in the same fiscal year.

- **Allocation–expenditure gap** (B − C) — difference between allocation and expenditure (positive = underspend; negative = overspend).
- **KRT share of UCS budget** (C/A, %) — proportion of UCS resources (defined as the total allocated budget under the UCS, excluding healthcare provider salaries, for the same fiscal year) actually spent on KRT.

The KRT scope covers costs of HD sessions, vascular access procedures, PD services, PD solutions and equipment, erythropoietin for all dialysis (HD+PD) patients, and kidney transplantation surgery and immunosuppressive therapy. These exclude not directly attributable to KRT e.g. medication other than EPO, treatment of other comorbidities, and inpatient/outpatient services for complications. All figures are program-level (not patient-linked) and reported in THB with consistent rounding; no inflation adjustment.

*5.6 Mortality analysis (system-level)*

We examined mortality among patients receiving dialysis between January 2018 and September 2024 using monthly administrative records.

Classification of deaths:

Monthly death counts were derived separately for HD and PD patients. Deaths were further classified into:

- *Early deaths*: occurring within 90 days of dialysis initiation.
- *Late deaths*: occurring ≥90 days after initiation.

This classification was applied to both HD and PD and combined at the system level to produce total early and late deaths.

Attribution of dialysis modality at death:

To avoid misclassification from short-term transfers prior to death (e.g., PD patients switched to HD during the terminal admission), deaths were attributed to each patient's *chronic modality* using the following rule:

- If a patient changed modality within 14 days before death, the death was attributed to the longer-term chronic mode used prior to transfer.
- Otherwise, the death was attributed to the last recorded modality.

This rule was applied consistently to both denominators and numerators in modality-specific analyses.

Mortality rates:

Mortality was assessed at the system level (all dialysis) and stratified by modality (HD, PD). Denominators were the number of active dialysis patients at the start of each month: total patients for system-level analyses, and modality-specific populations for HD and PD analyses. These denominators represent patient-months at risk. Monthly mortality rates were calculated as the number of deaths divided by the active patient count, expressed per 1,000 patient-months. Rates were scaled to 1,000 patient-years (×12) for summary comparisons with the epidemiologic literature. The proportion of early deaths among all deaths was also computed. Pre-policy (Jan 2018–Jan 2022) and post-policy (Feb 2022–Sep 2024) periods were compared descriptively.

Interrupted time-series (ITS) analysis

*(see section 5.3)*

*5.7 Patient-level analyses (incident cohort)*

We examined all incident dialysis patients initiating HD or PD between 1 January 2020 and 1 September 2024.

*Variables*

Baseline demographics included age, sex, region, and registration site. Regions were grouped into Bangkok, Central, North, Northeast, and South. Registration site type was classified as: (i) public hospital with in-house HD, (ii) public hospital with outsourced HD, or (iii) private hospital/clinic. Urban registration was defined as a hospital located in a provincial capital or municipality (thesaban nakhon / thesaban mueang), including Bangkok and Pattaya.

Comorbidities were derived from ICD-10 codes and summarised with the Charlson Comorbidity Index (CCI) (categories: 0–2, 3–4, 5–7, ≥8). Individual comorbidities were also reported.

*Dialysis modality*

- **Initial modality:** first treatment received (HD or PD).
- **Main modality at 90 days:** defined among patients alive and without transplantation at day 90. For those who died within 90 days, the last recorded modality was retained.

*Unplanned initiation*

Defined as initiation of KRT with HD using a non-tunnelled central venous catheter. This definition includes patients who ultimately received PD but required temporary HD at initiation.

*Vascular access*

Restricted to HD initiators from 1 January 2020 onward, when systematic recording became mandatory.

- **Initial access**: AVF/AVG, tunnelled catheter, or non-tunnelled catheter.
- **Access at 90 and 180 days**: assessed among patients alive on HD without transplantation.
- **Conversion to permanent access**: first use of AVF/AVG (primary definition); secondary definition included tunnelled catheter.
- **Time on any catheters**: interval from initiation to first AVF/AVG use.
- **Time on non-**tunnelled catheter: interval from initiation to first AVF/AVG/Tunnelled catheter use.

Statistical analysis

- Categorical variables: Pearson $\chi^2$ test.
- Normally distributed continuous variables: mean (SD), t-test.
- Non-normal variables (e.g. time to permanent access): median (IQR), Wilcoxon rank-sum test.

*Survival analysis*

Cox proportional hazards models estimated associations between policy period at initiation (post vs pre) and mortality. Models adjusted for unplanned initiation, age (continuous), sex, CCI category, region, and private-hospital registration.

Three prespecified models:

1. Overall mortality from day 0.
2. **Early mortality** (follow-up truncated at 90 days).
3. **Late mortality** (90-day landmark; risk set restricted to survivors at day 90).

Time was measured in years; ties handled with the Efron method. Adjusted hazard ratios (aHRs) with 95% CIs were reported.

*Cox proportional hazards models*

Assessed with Schoenfeld residuals and log(−log) survival plots. Given evidence of non-proportionality for the overall policy indicator, early and late windows were prespecified as primary.

### 6. Use of language models

Language assistance was provided using ChatGPT (GPT-5, OpenAI) to improve grammar, clarity, and style, and to provide limited coding support under full author supervision. Examples included refactoring plotting scripts and reformatting Stata/R code. The model was not used to generate, analyse, or interpret data, nor to perform statistical modelling. No patient-level or identifiable data were uploaded. All outputs were reviewed and verified by the authors, who take full responsibility for the content.

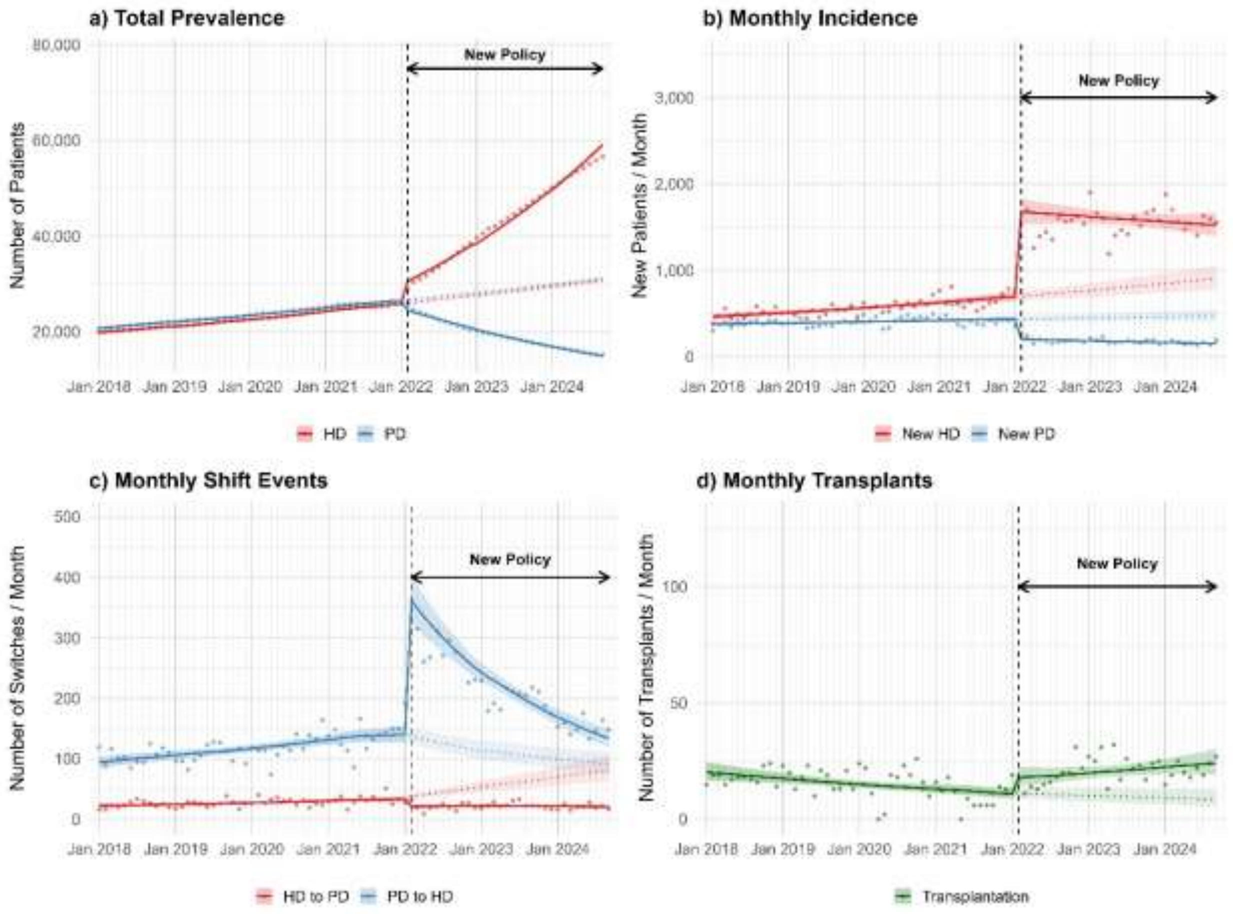


Supplementary Figure S1. Impact of the 2022 Policy on Absolute Patient Volumes.

(a) Total monthly prevalence (number of active patients) for haemodialysis (HD) and peritoneal dialysis (PD). (b) Total monthly incidence (number of new patient initiations). (c) Monthly frequency of modality switching events (number of patients switching modality). (d) Monthly frequency of kidney transplantations (number of procedures). Note: Values represent absolute counts (number of patients or events) rather than population-adjusted rates. Solid lines show observed data; dashed lines show fitted trends from negative binomial regression; dotted lines represent the counterfactual scenario (expected counts without policy intervention). Shaded ribbons indicate 95% confidence intervals. The vertical dashed line marks the policy implementation (Feb 2022). Abbreviations: HD, haemodialysis; PD, peritoneal dialysis.

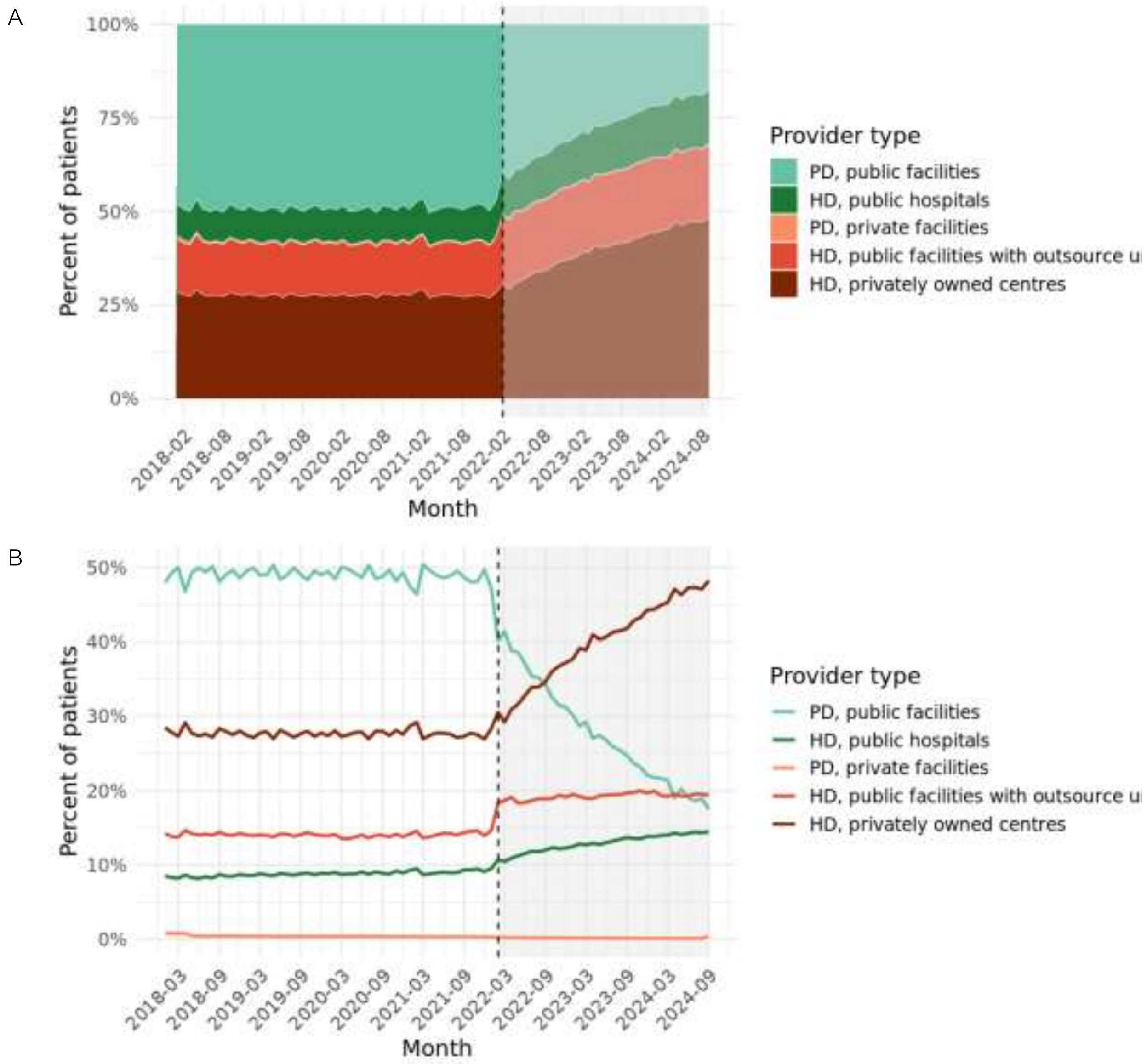


**Supplementary Figure S2.** Temporal trends in dialysis patient distribution by provider ownership type

(A) Monthly proportions of patients by provider ownership type, shown as stacked areas.

(B) Monthly proportions of patients by provider ownership type, shown as line trends.

Grey shading and dashed vertical line indicate the period after implementation of the February 2022 policy reform.

Supplementary Table S3. Impact of the 2022 Policy on Dialysis Service Utilisation, Modality Shifts, and Kidney Transplantation

| Outcome Metric | Pre-Policy Rate (Mean Annualised) | Pre-Policy Rate (Mean Annualised) | Immediate Policy Effect (Level change) IRR (95% CI) | Post-Policy trend (Slope change) IRR (95% CI) |
|---|---|---|---|---|
| Dialysis Prevalence *(per million UCS population)* | | | | |
| Total Dialysis Cases | 967.9 | 1322.4 | 1.02 (1.01–1.02)*** | 1.004 (1.004–1.005)*** |
| HD | 475.2 | 915.1 | 1.16 (1.14–1.17)* | 1.016 (1.015–1.017)*** |
| PD | 492.7 | 407.2 | 0.94 (0.93–0.95)* | 0.979 (0.979–0.980)*** |
| Dialysis Incidence *(per million UCS population)* | | | | |
| Total New Cases | 245.5 | 450.9 | 1.67 (1.50–1.87)* | 0.990 (0.985–0.996)*** |
| New HD | 143.5 | 405.8 | 2.42 (2.16–2.72)* | 0.989 (0.983–0.994)*** |
| New PD | 102.0 | 45.1 | 0.47 (0.42–0.53)* | 0.988 (0.983–0.994)*** |
| Modality Shifts *(per 100 patient-year; in origin mode)* | | | | |
| PD to HD | 6.0 | 13.7 | 2.68 (2.32–3.10)* | 0.981 (0.974–0.988)*** |
| HD to PD | 1.5 | 0.6 | 0.56 (0.46–0.70)* | 0.975 (0.965–0.985)*** |
| Kidney Transplantation | | | | |
| Transplantation Rate *(per 100 dialysis patient-year)* | 0.4 | 0.4 | 1.56 (1.13–2.16) | 1.019 (1.004–1.034)* |
| Transplants *(per million UCS population)* | 3.8 | 5.3 | - | - |

UCS population = mid-year number of universal coverage scheme beneficiaries, IRR = Incidence Rate Ratio. CI = Confidence Interval. Significance levels: * $p < 0.05$, ** $p < 0.01$, *** $p < 0.001$. All models adjusted for population growth (offset) or at-risk denominator (active dialysis population for shifts and transplantation)..

Supplementary Table S4. Kidney replacement therapy (KRT) expenditure and budget share, FY2018–FY2024.

| Fiscal year | Overall UHC budget (A) Billion THB (million USD) | Allocated KRT budget (B) Billion THB (million USD) | Actual KRT expenditure (C) Billion THB (million USD) | % increased from FY2018 | % KRT expense on Overall UHC budget (C/A) | Allocation–expenditure gap (B-C) Billion THB (million USD) |
|---|---|---|---|---|---|---|
| FY 2018 (Oct 17-Sep18) | 160.2 ($ 4,718.1) | 8.2 ($ 240.5) | 8.8 ($ 260.0) | (ref) | 5.5% | (-$ 19.6) |
| FY 2019 (Oct 18-Sep19) | 166.4 ($ 4,901.8) | 8.3 ($ 243.9) | 9.0 ($ 263.9) | +1.5% | 5.4% | (-$ 20.0) |
| FY 2020 (Oct 19-Sep20) | 173.8 ($ 5,116.9) | 9.4 ($ 276.1) | 10.4 ($ 304.9) | +17.3% | 6.0% | (-$ 28.8) |
| FY 2021 (Oct 22-Sep21) | 177.2 ($ 5,218.5) | 9.7 ($ 286.3) | 11.9 ($ 350.0) | +34.6% | 6.7% | (-$ 63.7) |
| FY 2022 (Oct 21-Sep22) | 158.3 ($ 4,661.8) | 9.7 ($ 286.6) | 12.4 ($ 364.7) | +40.2% | 7.8% | (-$ 78.1) |
| FY 2023 (Oct 22-Sep23) | 161.6 ($ 4,759.2) | 10.0 ($ 293.1) | 13.4 ($ 395.5) | +52.1% | 8.3% | (-$ 102.4) |
| FY 2024 (Oct 23-Sep24) | 165.5 ($ 4,874.7) | 12.8 ($ 377.2) | 15.4 ($ 452.1) | +73.9% | 9.3% | (-$ 74.9) |

All amounts are program-level, expressed in billion Thai Baht (THB) and million US Dollar(USD) using fixed foreign exchange rate [1 THB = 0.02945 USD]; Allocation–expenditure gap is calculated from allocated budget – actual expenditure (negative = overspend). Expenditure includes costs directly attributable to KRT (erythropoietin for dialysis patients, hemodialysis sessions, vascular access procedures, peritoneal dialysis services, PD solutions/equipment, kidney transplantation surgery and immunosuppressive therapy) and excludes treatment of other comorbidities, medications other than erythropoietin, and non-KRT inpatient/outpatient service.

Supplementary Table S5. Baseline characteristics of incident dialysis patients by policy period and main dialysis modality

| Main dialysis modality (at 90-day)† | Pre-policy (Jan 2020–Jan 2022) | | Post-policy (Feb 2022–Sep 2024) | |
|---|---|---|---|---|
| | HD N=9,447 | PD N=17,020 | HD N=47,179 | PD N=7,910 |
| *Demographic and clinical characteristics* | | | | |
| Age (years), mean ± SD | 60.6 ± 14.2 | 57.4 ± 13.5 | 60.4 ± 13.6 | 58.4 ± 14.3 |
| Age group, n(%) | | | | |
| <20 | 62 (0.7%) | 218 (1.3%) | 240 (0.5%) | 171 (2.2%) |
| 20-44 | 1,197 (12.7%) | 2,364 (13.9%) | 5,649 (12.0%) | 953 (12.0%) |
| 45-59 | 2,669 (28.3%) | 6,083 (35.7%) | 14,037 (29.8%) | 2,591 (32.8%) |
| 60-74 | 4,108 (43.5%) | 7,182 (42.2%) | 21,023 (44.6%) | 3,441 (43.5%) |
| 75+ | 1,404 (14.9%) | 1,170 (6.9%) | 6,209 (13.2%) | 754 (9.5%) |
| *missing* | 7 (0.1%) | 3 (0.0%) | 21 (0.0%) | 0 (0.0%) |
| Male sex, n (%) | 4,572 (48.4%) | 8,464 (49.7%) | 24,488 (51.9%) | 3,691 (46.7%) |
| *Missing* | 6 (0.1%) | 2 (0.0%) | 21 (0.0%) | 0 (0.0%) |
| Charlson Comorbidity Index, mean ± SD | 6.3 ± 2.3 | 5.8 ± 2.1 | 6.1 ± 2.2 | 6.0 ± 2.2 |
| Charlson Comorbidity Index category, n(%) | | | | |
| 0–2 | 467 (4.9%) | 1,166 (6.9%) | 2,756 (5.8%) | 480 (6.1%) |
| 3–4 | 1,637 (17.3%) | 3,319 (19.5%) | 8,150 (17.3%) | 1,413 (17.9%) |
| 5–7 | 4,698 (49.7%) | 9,137 (53.7%) | 24,100 (51.1%) | 4,137 (52.3%) |
| ≥8 | 2,631 (27.9%) | 3,395 (19.9%) | 12,131 (25.7%) | 1,880 (23.8%) |
| *missing* | 14 (0.1%) | 3 (0.0%) | 42 (0.1%) | 0 (0.0%) |
| Comorbidities (details) | | | | |
| Hypertension, n(%) | 8,786 (93.1%) | 16,319 (95.9%) | 42,940 (91.1%) | 7,474 (94.5%) |
| Diabetes mellitus, n(%) | 6,243 (66.2%) | 11,566 (68.0%) | 31,295 (66.4%) | 5,449 (68.9%) |
| History of myocardial infarction, n(%) | 1,162 (12.3%) | 1,763 (10.4%) | 4,991 (10.6%) | 948 (12.0%) |
| Congestive heart failure, n(%) | 3,326 (35.3%) | 5,572 (32.7%) | 15,196 (32.2%) | 2,607 (33.0%) |
| Peripheral vascular disease, n(%) | 380 (4.0%) | 475 (2.8%) | 1,657 (3.5%) | 257 (3.2%) |
| History of stroke/TIA, n(%) | 1,477 (15.7%) | 2,252 (13.2%) | 7,357 (15.6%) | 1,267 (16.0%) |
| Dementia, n(%) | 67 (0.7%) | 44 (0.3%) | 259 (0.5%) | 41 (0.5%) |
| Hemiplegia, n(%) | 378 (4.0%) | 563 (3.3%) | 1,865 (4.0%) | 350 (4.4%) |
| Liver disease (mild), n(%) | 699 (7.4%) | 1,179 (6.9%) | 3,608 (7.7%) | 632 (8.0%) |
| Liver disease (moderate to severe), n(%) | 104 (1.1%) | 148 (0.9%) | 426 (0.9%) | 81 (1.0%) |
| Chronic pulmonary disease, n(%) | 958 (10.2%) | 1,826 (10.7%) | 4,881 (10.4%) | 853 (10.8%) |
| Connective tissue disease, n(%) | 268 (2.8%) | 526 (3.1%) | 1,339 (2.8%) | 244 (3.1%) |

| Main dialysis modality (at 90-day)† | Pre-policy (Jan 2020–Jan 2022) | | Post-policy (Feb 2022–Sep 2024) | |
|---|---|---|---|---|
| | HD N=9,447 | PD N=17,020 | HD N=47,179 | PD N=7,910 |
| Peptic ulcer disease, n(%) | 513 (5.4%) | 947 (5.6%) | 2,891 (6.1%) | 428 (5.4%) |
| Any malignancy, n(%) | 646 (6.8%) | 487 (2.9%) | 2,357 (5.0%) | 275 (3.5%) |
| Metastatic solid tumor, n(%) | 87 (0.9%) | 40 (0.2%) | 318 (0.7%) | 21 (0.3%) |
| AIDs, n(%) | 0 (0.00%) | 0 (0.00%) | 0 (0.00%) | 0 (0.00%) |
| *Registration site* | | | | |
| Registered in urban area, n(%) | 8,677 (91.85%) | 15,032 (88.32%) | 42,856 (90.84%) | 6,845 (86.54%) |
| Region of registration, n (%) | | | | |
| • Bangkok | 1,455 (15.4%) | 606 (3.6%) | 4,740 (10.0%) | 276 (3.5%) |
| • Central | 1,842 (19.5%) | 2,904 (17.1%) | 7,504 (15.9%) | 1,168 (14.8%) |
| • North | 1,411 (14.9%) | 3,676 (21.6%) | 7,657 (16.2%) | 1,941 (24.5%) |
| • East | 1,084 (11.5%) | 1,283 (7.5%) | 4,837 (10.3%) | 521 (6.6%) |
| • Northeast | 2,769 (29.3%) | 6,678 (39.2%) | 18,551 (39.3%) | 2,780 (35.1%) |
| • South | 886 (9.4%) | 1,873 (11.0%) | 3,890 (8.2%) | 1,224 (15.5%) |
| *Dialysis initiation characteristics* | | | | |
| Unplanned dialysis initiation‡, n (%) | 5,483 (58.0%) | 6,271 (36.8%) | 30,825 (65.3%) | 2,302 (29.1%) |
| HD as first modality at initiation, n (%) | 9,447 (100.0%) | 6,383 (37.5%) | 47,179 (100.0%) | 2,428 (30.7%) |

† Main dialysis modality was defined using a 90-day ascertainment window. Patients were classified as HD if they initiated and remained on HD until 90 day, death or transplantation, and as PD if they initiated PD or transitioned from HD to PD within 90 days.

‡ Unplanned dialysis initiation was defined as HD initiation using a non-tunneled central venous catheter as the first modality.

Supplementary Table S6. Trends in initial vascular access and conversion to permanent access among incident HD patients, 2020–2024.

| Years of HD initiation | 2020<br>N=3,801 | 2021<br>N=4,611 | 2022*<br>N=17,666 | 2023*<br>N=17,756 | 2024*<br>N=12,275 | p-value† |
|---|---|---|---|---|---|---|
| Initial vascular access, n(%) | | | | | | <0.001 |
| AVF/AVG | 814 (21.4%) | 962 (20.9%) | 2,907 (16.5%) | 3,769 (21.2%) | 3,067 (25.0%) | |
| Tunnelled CVC | 856 (22.5%) | 887 (19.2%) | 2,606 (14.8%) | 2,461 (13.9%) | 1,611 (13.1%) | |
| Non-tunnelled CVC | 2,131 (56.1%) | 2,762 (59.9%) | 12,153 (68.8%) | 11,526 (64.9%) | 7,597 (61.9%) | |
| Conversion to permanent access | | | | | | |
| AVF/AVG use by day 90, n(%) | 2,082 (54.8%) | 2,560 (55.5%) | 9,438 (53.4%) | 9,841 (55.4%) | 7,277 (59.3%) | <0.001 |
| AVF/AVG use by day 180, n(%) | 2,485 (65.4%) | 3,003 (65.1%) | 11,817 (66.9%) | 12,220 (68.8%) | 8,814 (71.8%) | <0.001 |
| Days on any catheter, median (IQR) | 29.0 (0.0-106.0) | 29.0 (0.0-104.0) | 40.0 (5.0-127.0) | 34.0 (0.0-118.0) | 25.0 (0.0-98.0) | <0.001 |
| AVF/AVG or tunnelled CVC by day 90, n(%) | 2,830 (74.5%) | 3,427 (74.3%) | 13,475 (76.3%) | 13,914 (78.4%) | 9,862 (80.3%) | <0.001 |
| AVF/AVG or tunnelled CVC by day 180, n(%) | 3,015 (79.3%) | 3,678 (79.8%) | 14,988 (84.8%) | 15,245 (85.9%) | 10,723 (87.4%) | <0.001 |
| Days on non-tunnelled catheter, median (IQR) | 0.0 (0.0-32.0) | 4.0 (0.0-38.0) | 11.0 (0.0-46.0) | 8.0 (0.0-41.0) | 7.0 (0.0-38.0) | <0.001 |

Abbreviations: AVF, arteriovenous fistula; AVG, arteriovenous graft; CVC, central venous catheter; IQR, interquartile range.

The vascular access cohort included incident patients with hemodialysis established as the main modality at day 90. Patients who switched to peritoneal dialysis or underwent kidney transplantation within 90 days were excluded, while those who died within 90 days were retained. Analysis was restricted to the 2020–2024 period due to the commencement of systematic vascular access recording.

† P-values were derived from Pearson's chi-squared test for categorical comparisons and from the Kruskal-Wallis test for continuous measures.

Supplementary Table S7. Interrupted time series (ITS) analysis of dialysis mortality before and after the 2022 policy

| Population / Outcomes | Baseline trend IRR per month (95% CI) | p-value | Immediate level change (policy impact) IRR (95% CI) | p-value | Post-Policy Trend IRR per month (95% CI) | p-value |
|---|---|---|---|---|---|---|
| All dialysis | 1.004 (1.002 – 1.006) | < 0.001 | 1.19 (1.09-1.30) | < 0.001 | 0.985 (0.981 – 0.990) | < 0.001 |
| – Early mortality (<90 d) | 1.002 (0.999 – 1.005) | 0.08 | 1.77 (1.59-1.97) | < 0.001 | 0.992 (0.987-0.997) | 0.002 |
| – Late mortality (≥90 d) | 1.004 (1.003-1.007) | < 0.001 | 1.07 (0.98-1.16) | 0.13 | 0.984 (0.980-0.989) | < 0.001 |
| Haemodialysis (HD) | 1.006 (1.003-1.008) | < 0.001 | 1.45 (1.30-1.60) | < 0.001 | 0.979 (0.975-0.985) | < 0.001 |
| – Early mortality (<90 d) | 1.005 (1.001-1.009) | 0.004 | 1.62 (1.44-1.83) | < 0.001 | 0.988 (0.982-0.993) | < 0.001 |
| – Late mortality (≥90 d) | 1.006 (1.003-1.008) | < 0.001 | 1.21 (1.08-1.35) | 0.001 | 0.982 (0.976 – 0.987) | < 0.001 |
| Peritoneal dialysis (PD) | 1.004 (1.002-1.005) | < 0.001 | 0.97 (0.89-1.05) | 0.44 | 0.994 (0.990-0.998) | 0.005 |
| – Early mortality (<90 d) | 0.999 (0.996-1.003) | 0.87 | 1.15 (0.94-1.40) | 0.18 | 0.998 (0.988-1.008) | 0.74 |
| – Late mortality (≥90 d) | 1.004 (1.002 – 1.005) | < 0.001 | 0.96 (0.88-1.04) | 0.27 | 0.994 (0.990-0.998) | 0.002 |

Segmented interrupted time series (ITS) analyses encompass 81 months (January 2018–September 2024). Early mortality is death within the first 90 days of dialysis; late mortality is death at ≥90 days. To minimize bias from terminal transfers, Modality at Death was attributed to the chronic modality if utilized for ≥ 14 days prior to death; otherwise, it was attributed to the most recent modality. Baseline Trend denotes pre-policy monthly change (pre-February 2022). Immediate level change represents the step-change in mortality risk at implementation. Post-Policy Trend indicates the subsequent monthly change in risk.

Supplementary Table S8. Multivariable Cox models for mortality among incident dialysis patients

| Covariates | Overall deaths aHR (95% CI) | P value | Early deaths (<90d) aHR (95% CI) | P value | Late deaths (≥90d) aHR (95% CI) | P value |
|---|---|---|---|---|---|---|
| Policy period: post-policy (vs pre-policy) | 1.40 (1.37-1.43) | < 0.001 | 1.38 (1.32-1.45) | < 0.001 | 1.40 (1.36-1.44) | < 0.001 |
| Unplanned initiation | 1.30 (1.27-1.33) | < 0.001 | 2.30 (2.20-2.41) | < 0.001 | 1.08 (1.05-1.11) | < 0.001 |
| Age at start (per year) | 1.02 (1.02-1.02) | < 0.001 | 1.03 (1.02-1.03) | < 0.001 | 1.01 (1.01-1.01) | < 0.001 |
| Female (vs male) | 0.97 (0.95-0.99) | 0.002 | 0.96 (0.92-0.99) | 0.026 | 0.97 (0.95-0.99) | 0.012 |
| Charlson comorbidity index (vs 0–2) | | | | | | |
| 3–4 | 1.62 (1.50-1.76) | < 0.001 | 1.76 (1.46-2.12) | < 0.001 | 1.62 (1.48-1.77) | < 0.001 |
| 5–7 | 2.43 (2.24-2.62) | < 0.001 | 2.25 (1.87-2.70) | < 0.001 | 2.55 (2.33-2.78) | < 0.001 |
| ≥8 | 3.63 (3.34-3.94) | < 0.001 | 3.45 (2.86-4.17) | < 0.001 | 3.76 (3.43-4.12) | < 0.001 |
| Region of registration (vs Bangkok) | | | | | | |
| Central | 1.16 (1.12-1.22) | < 0.001 | 1.27 (1.17-1.39) | < 0.001 | 1.13 (1.07-1.18) | < 0.001 |
| North | 1.08 (1.04-1.13) | < 0.001 | 1.11 (1.02-1.21) | 0.02 | 1.07 (1.02-1.13) | 0.008 |
| East | 1.03 (0.98-1.08) | 0.32 | 1.07 (0.97-1.18) | 0.19 | 1.01 (0.95-1.07) | 0.82 |
| Northeast | 1.15 (1.10-1.19) | < 0.001 | 1.26 (1.17-1.36) | < 0.001 | 1.10 (1.05-1.15) | < 0.001 |
| South | 1.17 (1.12-1.23) | < 0.001 | 1.19 (1.08-1.32) | < 0.001 | 1.16 (1.10-1.23) | < 0.001 |

Complete case analysis (N = 102,868). Adjusted hazard ratios (aHRs) with 95% confidence intervals were estimated using Cox proportional hazards models (Efron method for ties; time in years, scale = 365.25). Three models are shown: (i) Overall – time zero at dialysis initiation, failure = all-cause death; (ii) Early <90 days – follow-up truncated at 90 days; and (iii) Late ≥90 days – risk set includes patients alive at day 90, with time counted from that landmark.

Supplementary Table S9. Sensitivity analysis of multivariable Cox models for mortality among incident HD patients considering conversion to permanent vascular access use by 90-day

| Covariate (reference) | Overall deaths aHR* (95% CI) | P value | Early deaths (<90d) aHR (95% CI) | P value | Late deaths (≥90d) aHR (95% CI) | P value |
|---|---|---|---|---|---|---|
| Policy period: post-policy (vs pre-policy) | 1.53 (1.48–1.59) | < 0.001 | 1.05 (0.99-1.01) | 0.09 | 2.01 (1.91-2.11) | < 0.001 |
| AVF/AVG/Tunnelled catheter use by day 90 | 0.34 (0.33-0.35) | < 0.001 | 0.11 (0.11-0.12) | < 0.001 | 0.83 (0.79-0.87) | < 0.001 |

Complete case analysis (N = 65,381). Adjusted hazard ratios (aHRs) with 95% confidence intervals were estimated using Cox proportional hazards models (Efron method for ties; time in years, scale = 365.25) adjusted by unplanned initiation, age, sex, Charlson's comorbidity index category, and regions. Three models are shown: (i) Overall – time zero at dialysis initiation, failure = all-cause death; (ii) Early <90 days – follow-up truncated at 90 days; and (iii) Late ≥90 days – risk set includes patients alive at day 90, with time counted from that landmark.

Strengthening the reporting of observational studies in epidemiology (STROBE) checklist

| Item | Item No | Recommendation | Page No |
|---|---|---|---|
| Title and abstract | 1 | (*a*) Indicate the study's design with a commonly used term in the title or the abstract | 1-2 |
| | | (*b*) Provide in the abstract an informative and balanced summary of what was done and what was found | 2-3 |
| Introduction | | | |
| Background/rationale | 2 | Explain the scientific background and rationale for the investigation being reported | 4-6 |
| Objectives | 3 | State specific objectives, including any prespecified hypotheses | 5-6 |
| Methods | | | |
| Study design | 4 | Present key elements of study design early in the paper | 6 |
| Setting | 5 | Describe the setting, locations, and relevant dates, including periods of recruitment, exposure, follow-up, and data collection | 6-7 |
| Participants | 6 | (*a*) Give the eligibility criteria, and the sources and methods of selection of participants. Describe methods of follow-up | 7-8 |
| Variables | 7 | Clearly define all outcomes, exposures, predictors, potential confounders, and effect modifiers. Give diagnostic criteria, if applicable | 9-13 |
| Data sources/ measurement | 8* | For each variable of interest, give sources of data and details of methods of assessment (measurement). Describe comparability of assessment methods if there is more than one group | 7-12 |
| Bias | 9 | Describe any efforts to address potential sources of bias | 12 |
| Study size | 10 | Explain how the study size was arrived at | 8 |
| Quantitative variables | 11 | Explain how quantitative variables were handled in the analyses. If applicable, describe which groupings were chosen and why | 7-12 |
| Statistical methods | 12 | (*a*) Describe all statistical methods, including those used to control for confounding | 8-12 |
| | | (*b*) Describe any methods used to examine subgroups and interactions | 8-12 |
| | | (*c*) Explain how missing data were addressed | 12 |
| | | (*d*) If applicable, explain how loss to follow-up was addressed | N/A |
| | | (*e*) Describe any sensitivity analyses | 8-12 |
| Results | | | |
| Participants | 13* | (a) Report numbers of individuals at each stage of study—eg numbers potentially eligible, examined for eligibility, confirmed eligible, included in the study, completing follow-up, and analysed | 13 |
| | | (b) Give reasons for non-participation at each stage | N/A |
| | | (c) Consider use of a flow diagram | N/A |

| Item | Item No | Recommendation | Page No |
| --- | --- | --- | --- |
| Descriptive data | 14* | (a) Give characteristics of study participants (eg demographic, clinical, social) and information on exposures and potential confounders | 14-15 |
| | | (b) Indicate number of participants with missing data for each variable of interest | 14.-15 ,Table1 |
| | | (c) Summarise follow-up time (eg, average and total amount) | 14-15 |
| Outcome data | 15* | Report numbers of outcome events or summary measures over time | Table 2 |
| Main results | 16 | (*a*) Give unadjusted estimates and, if applicable, confounder-adjusted estimates and their precision (eg, 95% confidence interval). Make clear which confounders were adjusted for and why they were included | Table 2 |
| | | (*b*) Report category boundaries when continuous variables were categorized | Table1 |
| | | (*c*) If relevant, consider translating estimates of relative risk into absolute risk for a meaningful time period | N/A |
| Other analyses | 17 | Report other analyses done—eg analyses of subgroups and interactions, and sensitivity analyses | N/A |
| Discussion | | | |
| Key results | 18 | Summarise key results with reference to study objectives | 17 |
| Limitations | 19 | Discuss limitations of the study, taking into account sources of potential bias or imprecision. Discuss both direction and magnitude of any potential bias | 20-21 |
| Interpretation | 20 | Give a cautious overall interpretation of results considering objectives, limitations, multiplicity of analyses, results from similar studies, and other relevant evidence | 17-20 |
| Generalisability | 21 | Discuss the generalisability (external validity) of the study results | 20-21 |
| Other information | | | |
| Funding | 22 | Give the source of funding and the role of the funders for the present study and, if applicable, for the original study on which the present article is based | 22 |

(END)
